# Structures and Bioactivities of Steroidal Saponins Isolated from the Genera *Dracaena* and *Sansevieria*

**DOI:** 10.3390/molecules26071916

**Published:** 2021-03-29

**Authors:** Zaw Min Thu, Sann Myint Oo, Thinn Myat Nwe, Hnin Thanda Aung, Chabaco Armijos, Faiq H. S. Hussain, Giovanni Vidari

**Affiliations:** 1 Department of Chemistry, Kalay University, Kalay 03044, Myanmar; sannmyintoo@kalayuniversity.edu.mm (S.M.O.); thinnmyatnwe@kalayuniversity.edu.mm (T.M.N.); 2 Department of Chemistry, University of Mandalay, Mandalay 100103, Myanmar; hninthandaaung@mu.edu.mm; 3 Departamento de Química y Ciencias Exactas, Universidad Técnica Particular de Loja, San Cayetano Alto s/n, Loja 1101608, Ecuador; 4 Medical Analysis Department, Faculty of Science, Tishk International University, Erbil 44001, Iraq; faiq.hussain@ishikuniversity.onmicrosoft.com

**Keywords:** *Dracaena*, *Sansevieria*, phytochemical constituents, biological activities, steroidal saponins, cytotoxicity

## Abstract

The species *Dracaena* and *Sansevieria*, that are well-known for different uses in traditional medicines and as indoor ornamental plants with air purifying property, are rich sources of bioactive secondary metabolites. In fact, a wide variety of phytochemical constituents have been isolated so far from about seventeen species. This paper has reviewed the literature of about 180 steroidal saponins, isolated from *Dracaena* and *Sansevieria* species, as a basis for further studies. Saponins are among the most characteristic metabolites isolated from the two genera. They show a great variety in structural motifs and a wide range of biological activities, including anti-inflammatory, anti-microbial, anti-proliferative effects and, in most case, remarkable cytotoxic properties.

## 1. Introduction

The dracaenoid genera *Dracaena* and *Sansevieria* are differentiated in the APG IV system of flowering plant classification and are placed in the subfamily, Nolinoideae of the family Asparagaceae of monocotyledons, in the order Asparagales [1]. However, recent molecular phylogenetic studies showed that *Sansevieria* was nested within *Dracaena*, rendering the latter paraphyletic, unless *Dracaena* was expanded to include species formerly placed in *Sansevieria* [2,3,4]. In this paper, we have maintained the historical division in the two genera because the chemical literature is mainly based on the former botanical classification.

The genus *Dracaena* comprises more than 110 accepted species. They are mostly succulent shrubs and trees that are mainly distributed in Africa, Australia, India, and Southeast Asia [5]. *Dracaena* species are widely used in various traditional medicine to treat different diseases, such as hemorrhoids and infections [6,7]. Dragon’s blood is a deep red resin with great commercial value that is obtained from cut leaves, stems and roots from different plant taxa including about six *Dracaena* plants that grow in China, Southeast Asia, West Africa, Arabian Peninsula, Yemen, India, and Macaronesia [8,9]. The resin has remarkable anti-inflammatory and antioxidant effects and it has widely been used in herbal medicines through the world for thousands of years as an efficacious remedy for the treatment of hemorrhage, dysentery, diarrhea, stomach and external ulcers, wounds, leucorrhea, rheumatism, fractures, piles, diabetes, and even tumors [9,10,11,12,13].

The genus *Sansevieria* comprises about 70 herbaceous species with rhizomatous roots, with a distribution ranging from Africa through Asia to Myanmar and the Islands of the Indian Ocean [14,15]. Leaves and rhizomes are used in folk medicine for treating asthma, cough, sexual weakness, hypertension, diarrhea, hemorrhoids, abdominal pains, colics, eczema, piles, edema, jaundice, anuria, palpitations, viral hepatitis, malaria, snake- and insect bites, etc. [16,17,18,19]. In addition to usages as herbal remedies, several *Dracaena* and *Sansevieria* species have great horticultural importance and are commercialized for use in landscaping and as indoor ornamental plants [20]. Moreover, it has been reported that *Dracaena* spp. can be used as bioindicators for the control of increasing air pollution in urban cities [21], whereas the leaf fiber of many *Sansevieria* are used for making fine matting, rope or cordage, and articles of clothing [14].

The most characteristic secondary metabolites isolated from *Dracaena* and *Sansevieria* are steroids, flavonoids, stilbenes, and saponins; flavonoids and saponins form the largest groups. The structures and bioactivities of phenolic constituents, flavonoids and stilbenoids have been reported in recent reviews [8,22]. On the other hand, there is great interest in saponins due to their intriguing structures and the potent analgesic, anti-inflammatory, antimicrobial, antioxidant, antiproliferative, hypocholesterolemic, and cytotoxic activities [23,24,25]. This paper describes the about 180 different saponins isolated from 14 *Dracaena* and 3 *Sansevieria* species that have been investigated so far for these metabolites. Biological activities have been summarized at the end of the paper. Most structures have been determined in the last twenty years. The literature has been retrieved from the databases Reaxys^®^, Scifinder^®^, and Google Scholar until the end 2020. A partial list of saponins isolated from *Dracaena* and *Sansevieria* species has been included in an earlier review on the Agavaceae family [23].

## 2. General Aspects of Steroidal Saponins Isolated from *Dracaena* and *Sansevieria* Species

**Figure 1:**
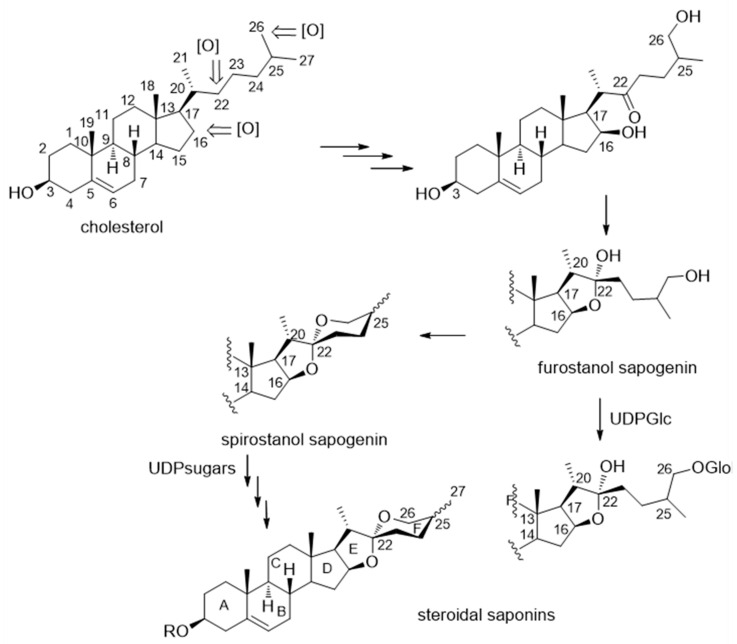
Main steps in the biosynthetic transformation of cholesterol to steroidal saponins.

Saponins are a group of plant glycosides characterized by their high surfactant properties. Therefore, they form stable soap-like foams in aqueous solution and are highly toxic if injected into the bloodstream because of powerful hemolytic effects, due to their capacity to alter membrane permeability. Saponins are classified as steroidal or triterpenoid saponins depending on the nature of the aglycone, called sapogenin. Steroidal glycosides [24], as those found in *Dracaena* and *Sansevieria* species, are naturally occurring sugar conjugates of a steroid core. Spirostanol and furostanol saponins are largely predominant in *Dracaena* and *Sansevieria* species. Their sapogenin cores are those of C_27_ sterols, biogenetically derived from cholesterol, in which oxidations of carbons C-16, C-22 and C-26 give rise to either a spiroacetal (spirostanol sapogenins) or a hemiacetal moiety (furostanol sapogenins), as shown in Figure 1. Thus, the spirostanol skeleton has a tetrahydrofuran ring (E) and a tetrahydropyran ring (F) joined at C-22 to give a spirane moiety, whereas the furostanol skeleton contains a tetrahydrofuran ring (E).

Other modifications of the steroid skeleton involve oxidation at C-1 (e.g., compound **1**), C-23 (e.g., **15**), C-24 (e.g., **9**), C-25 (e.g., **119**) and, less frequently, oxidation at C-4 (e.g., **137**), C-5 (e.g., **177**), C-6 (e.g., **24**), C-7 (e.g., **5**), C-12 (e.g., **23**), C-14 (e.g., **92**), C-17 (e.g., **7**), C-23 (e.g., **177**) and C-27 (e.g., **96**), introduction of a double bond between C-25 and C-27 (e.g., **1**) and, more rarely, between C-20 and C-22 (e.g., **140**). Furostanol acetals bearing a methoxy group at C-22 (e.g., **133**) are usually considered artifacts generated from the corresponding 22-OH derivatives as a result of extraction and processing of the plant materials with MeOH [24]. Rare C_27_ cholestane derivatives (e.g., **176**), as well as C_22_ (e.g., **165**) and C_21_ (e.g., **168**) pregnane steroidal sapogenins, arising from the oxidative rupture of the C-22/C-23, and C-20/C-22 bonds, respectively, of a cholestane precursor have also been isolated from a few *Dracaena* and *Sansevieria* species.

In relation to the stereochemistry of the sapogenin nucleus, the C-3, C-8, C-9, C-10, C-13, C-14, C-17, and C-20 centers retain the same stereochemistry as the parent cholesterol almost invariably. Whereas, the configurations of oxygenated substituents at C-1, C-16, and C-22 are *R*, *S*, and *R*, respectively, with the exception of saponin **90** that is 22*S*. C-23 stereocenters bearing a OH have the *R* configuration, while the C-24 and C-25 stereocenters may have either the *R* or *S* configuration. A mixture of stereoisomers can also occur in the same plant.

### 2.1. Glycosidic Moieties of Dracaena and Sansevieria Saponins

The glycone parts of *Dracaena and Sansevieria* saponins can be mono-, di-, tri- and tetra-glycosides, depending on the number of sugar units that may include β-D-glucopyranosyl (β-D-Glc*p*), α-L-rhamnopyranosyl (α-L-Rha*p*), α-L-arabinopyranosyl (α-L-Ara*p*), β-D-xylopyranosyl (β-D-Xyl*p*), and β-D-fucopyranosyl (β-D-Fuc*p*) moieties (Table 1).

One saponin (**48**) contains a β-D-apiofuranosyl (β-D-Api*f*) unit and four compounds (**23**, **73**–**75**) contain β-D-galactopyranosyl (β-D-Gal*p*) residues. Noteworthy, no furostanol monoglycoside has been isolated so far from *Dracaena* and *Sansevieria* species while also spirostanol monoglycosides are rare. Spirostanol di- and triglycosides, as well as furostanol tetraglycosides, are the most abundant saponins in *Dracaena* and *Sansevieria*. The sugar moieties can be present at one (monodesmosidic saponins) or two different positions (bisdesmosidic saponins) of the sapogenin core. One rare tetradesmodic saponin (**152**) is also known. Tri- and tetraglycoside units are branched with the exception of the linear triglycosides **73**–**75**.

Sugar units are attached through an acetal linkage to a hydroxyl group on the steroid core. The most common glycosylation sites are 1-OH, 3-OH, and 24-OH in spirostanes, and 1-OH, 3-OH, and 26-OH in furostanes (Table 1). The hydroxyl groups, 24-, 25- and 26-OH are usually monoglycosylated, whereas a branched oligosaccharide moiety is commonly attached to the other sites. An α-L-arabinopyranosyl residue is commonly attached to 1-OH, whereas 3-OH and 26-OH are mainly β-D-glucosylated; a β-D-fucopyranosyl moiety is the typical substituent of 24-OH (Table 1). In branched oligosaccharides, an α-L-rhamnopyranosyl group is almost invariably attached to 2′-OH, whereas a β-D-xylopyranosyl unit, an α-L-rhamnopyranosyl group or, less frequently, a β-D-glucopyranosyl residue is bonded to 3′-OH. An α-L-rhamnopyranoside moiety is also the common substituent at 4′-OH. Interestingly, β-D-fucopyranose and α-L-arabinopyranose, except for saponin **84**, have never been found as inner components of branched oligosaccharides in *Dracaena* and *Sansevieria* saponins. β-D-Galactopyranose has been identified as the terminal sugar of linear trisaccharide moieties (e.g., **74**).

Acetyl (e.g., **15**) and, more rarely, sulfatyl (e.g., **6**) groups may be present as part of the sugar moieties.

### 2.2. Isolation and Structure Determination

There is not a general specific procedure for the isolation of steroidal saponins from *Dracaena* and *Sansevieria*; instead, methods are analogous to those used for other natural saponins [24], for example from *Agave* species [25]. Based on our personal experience and key references in the literature [26,27,28,29,30,31,32,33], some general guidelines can, however, be suggested. Soon after collection, vegetal material is quickly air-dried in the shade to avoid enzymatic or microbial degradation and is then minced and cold- or heat-extracted by maceration or in a Soxhlet with MeOH or EtOH, or with 40–70% aqueous alcohol. The vegetal material is often defatted with a hydrocarbon solvent before the extraction with alcohol. Using MeOH, 22-OH furostanol glycosides are usually converted into their respective 22-methoxyl derivatives [31]. The presence of spirostanol and furostanol glycosides in the extract can be revealed by yellow spots on TLC plates sprayed with 1% anisaldehyde and 10% sulfuric acid in MeOH, followed by heating at 100 °C. Moreover, furostanol glycosides stain red with the Ehrlich reagent (1% 4-dimethylamino benzaldehyde and 10% HCl in MeOH), while spirostanol glycosides are colorless. To our knowledge, hyphenated techniques such as HPLC coupled with mass spectrometry (LC-MS) or with nuclear magnetic resonance spectroscopy (LC-NMR), and capillary electrophoresis coupled with mass spectroscopy (CEMS), have not been used until now for the rapid screening of the extracts/fractions for the presence and identification of saponins from *Dracaena* and *Sansevieria*.

Saponins may sometimes be precipitated from the crude extract by adding an excess of acetone, but usually the extract, after filtration, is carefully concentrated under reduced pressure in a rotary evaporator, paying attention to the inevitable formation of troublesome foams. To avoid excessive foaming and bumping, the rotation speed of the evaporation flask shall be moderate, and the use of a splash head and a vacuum regulator is highly recommended. The syrupy residue is then suspended in water and partitioned directly with *n*-BuOH or, for a preliminary defatting and fractionation, with hexane or Et_2_O or CH_2_Cl_2_, followed by EtOAc and *n*-BuOH, in the order. Saponins usually migrate to the *n*-BuOH soluble fraction; however, some amounts of the less polar monoglycosyl derivatives can also be extracted by EtOAc, while the most polar glycosides may remain in the aqueous layer. After concentration of these extracts under reduced pressure, the separation of a mixture of polar saponins into individual components is usually tedious and highly challenging. The traditional purification and separation procedures generally consist of fractionation over Sephadex LH-20 columns, followed by repetitive separations on silica gel columns using chloroform-methanol and/or chloroform-methanol-water in various ratios as eluents. Chromatographic separations on reverse-phase (e.g., RP-18 or Diaion^®^ HP-20) columns using a gradient of H_2_O–MeOH or H_2_O–MeCN as eluent are often performed. Preparative or semipreparative MPLC or even HPLC using analytical columns, and/or preparative TLC, are usually applied in the final steps of the purification process. Droplet countercurrent chromatographic (DCCC) techniques often give excellent separations, although the development of optimal conditions may be troublesome. At the end of the purification process, an isolated crystalline compound should be recrystallized, whenever possible.

Subsequently, the structure of a homogeneous saponin is established by a combination of chemical and spectroscopic methods [24,25,26,27,28,29,30,31,32,33]. Chemical reactions are sample demanding and usually require tens of milligrams of a saponin, while spectra can be determined on a few milligrams of a substance.

For a known saponin or sapogenin, comparison of the NMR spectra, melting point, and specific rotation with the literature is generally adequate to establish the identity. However, care must be used on comparing NMR spectra which have been determined in different solvents, as significant differences may exist. In fact, there is no general consensus on the best solvent to use, so that pyridine-*d*_5_, methanol-*d*_4_ and DMSO-*d*_6_ are all used indifferently.

The procedure for establishing the structure of a novel saponin usually involves the determination of: (i) The structures of sugar residues and the steroid aglycone; (ii) the sugar sequence; (iii) the glycosylation pattern; (iv) molecular stereochemistry.

Hydrolysis of the saponin under strong acid conditions (1N HCl, MeOH or dioxane–H_2_O, 1:1, 0.5–1 mg/mL, 80–90 °C, h; or 2 N aqueous CF_3_COOH, 3 mg/5 mL, 95 °C, 3 h) allows for the identification of the aglycone and the monosaccharide constituents separately. However, the possibility that the hydrolysis products are not natural sapogenins but are artifacts, should be considered. Individual monosaccharides and their ratio in the saponin are determined by TLC (anisaldehyde/sulfuric acid, followed by heating for the spot visualization), (enantioselective) GLC (alditol acetates or trifluoroacetates/TMS derivatives), (enantioselective) HPLC with a Refractive Index detector, optical rotation, and comparison with authentic samples. Partial hydrolysis (e.g., by 0.2 N HCl, dioxane–H_2_O, 1:1, 95 °C, 30 min–2 h), followed by isolation and characterization of prosapogenin and oligosaccharides, is employed for the determination of terminal sugars and sugar sequences [24]. A β-glucosidase enzyme can also be employed to hydrolyze the β-glucosidic linkage(s) of a glucoside. Interestingly, cleavage of a furostanol 26-*O*-glucoside by a furostanol glycoside 26-*O*-β-glucosidase affords directly the corresponding spirostane and β-D-glucose [24]. When acetyl or sulfate groups are present on the sugar moieties of a saponin, acetates are cleaved by treatment with 5% NaOMe or K_2_CO_3_ in MeOH or 10% ammonia solution in MeOH, whereas sulfate groups, identifiable by a strong S=O band at 1230 cm^−1^, can be detached by refluxing with a mixture of pyridine and dioxane [24]. The sites of a sapogenin where different sugar units are attached can be revealed by permethylation of the glycoside, followed by hydrolysis and identification of the partially methylated sugars and sapogenin by GLC and NMR spectroscopy, respectively. Hydrolysis of a permethylated saponin, followed by GC-MS analysis of the methylated sugar mixture after reduction (NaBH_4_) and acetylation (Ac_2_O/Py), is used for determining interglycosidic linkages.

As regards spectrometric methods, they are powerful and indispensable tools for the structure elucidation of saponins. Different ion peaks appearing in spectra obtained by soft-ionizing methods, such as FD-MS, ESI-MS, FAB-MS, (ESI-TOF)-MS in conjunction with multi-stage tandem mass spectrometry, not only provide the correct molecular weight but also, in many instances, the sequence of sugars in the oligosaccharide moiety, through study of the fragmentation pathways. Both negative and positive ion modes have been used to obtain the molecular weight of saponins. The loss of various monosaccharide units from a saponin molecule generates fragmentation peaks with a characteristic *m*/*z* value. Therefore, loss of fragments of 132, 146 and 162 mass units indicates cleavage of pentose (arabinose or xylose), deoxyhexose (rhamnose or fucose) and hexose (glucose or galactose) moieties, respectively. Of course, the information inferred from MS methods must be used in conjunction with ^1^H and ^13^C NMR spectroscopy (glycosidation and esterification shift rules, comparison of NMR data, utilization of the *J*^3^ values between H-1 and H-2 for determining the anomeric configuration) together with chemical strategies (see above) [24]. In fact, modern NMR techniques are the methods of choice for establishing the structure of the aglycone, the nature and number of the constituent sugar units including their ring sizes, anomeric configurations, interglycosidic linkages as well as the point(s) of attachment of the sugar chain to the sapogenin. Determination of all standard 1D and 2D ^1^H and ^13^C-NMR spectra are usually necessary to firmly establish the structure of a new saponin.

The most characteristic spectroscopic data of saponins are briefly summarized below. In addition, the consultation of the reference literature [24,25,34,35] is highly recommended.

### 2.3. NMR Spectra of Dracaena and Sansevieria Saponins

In the ^1^H NMR spectra of saponins, two singlets and two doublets occurring in the range of *δ* 0.5–1.7 are diagnostic for the methyl groups at C-10, C-13, C-20, and C-25 of a steroid nucleus. In the lower part of the same NMR region, an additional doublet can be attributed to the methyl group of a 6-deoxy-sugar residue, such as a rhamnosyl unit.

#### 2.3.1. Type of Parent Skeleton and Stereochemistry at C-22

The chemical shift of C-22 in the ^13^C NMR spectrum is indicative of the type of parent skeleton, i.e., spirostanol or furostanol. In the case of spirostanol glycosides, C-22 usually resonates below *δ*c 110 and, in the presence of C-23 and C-24 hydroxyls, the C-22 signal is shifted to lower field by 2–4 ppm. In furostanol glycosides having a free hydroxyl or a methoxyl group at C-22, the chemical shift of C-22 is about *δ*c 111 in the former case, and *δ*c 112–113 in the latter [25,34,35]. Moreover, furostane saponins do not exhibit IR absorptions at 920–915 and 900 cm^−1^ that are characteristic of the spiroketal moiety of a spirostane derivatives. The 920 cm^−1^ band is more intense than the 900 cm^−1^ one in (22*S*)-spirostanes; moreover, an intense band at 900 cm^−1^, compared to the 920 cm^−1^ band, is indicative of a (22*R*)-stereocenter. In addition, the 22*R* stereochemistry of spirostanes is deduced from the presence of two well separated H_2_-26 proton signals that appear at ~*δ* 4.05 (H-26_eq_), and ~3.35 (H-26_ax_), respectively.

#### 2.3.2. Stereochemistry at C-25

^1^H NMR spectra give information on the stereochemistry of the methyl group at C-25, that can occur as 25*R*- and 25*S* in steroidal saponins. Therefore, the signal of H_3_-27 usually occurs at *δ* 0.55–0.85 and *δ* 0.95–1.15 in (25*R*)- and (25*S*)-spirostanes, respectively. Moreover, the difference between the chemical shifts of axial and equatorial protons attached to C-23, C-24, and C-26 can be used to resolve the absolute configuration of C-25 in spirostanes. The differences *δ*eq − *δ*ax for H_2_-23, -24, -26 are usually >0.35 for the 25*S* configuration, while they are <0.20 ppm for (25*R*) compounds. ^13^C NMR spectrometry is also very informative about the stereochemistry at C-25. Highly diagnostic are the C-23 and C-25 signals that are significantly moved downfield (*δ*_C_ > 30.0) in (25*R*) stereoisomers. NOE and NOESY techniques are also used to determine the stereochemistry at position 25 in spirostanes through the NOE correlations between H-25 and 27-Me with adjacent protons on the ring [31]

For furostanol glycosides, the difference between the chemical shifts of the geminal H_2_-26 is < 0.48 for (25*R*) and > 0.57 for (25*S*) configuration. Moreover, the methyl group at C-25 resonates at *δ* 0.90–1.05 and at *δ* 0.95–1.10 for (25*R*)– and (25*S*)–stereoisomers, respectively [36,37,38,39].

The conversion of a furostanol saponin to the corresponding spirostanol form by hydrolysis or enzymatic cleavage of the sugar (usually glucose) moiety at C-26 is still the most reliable method for the prediction of the C-25 configuration in furostanol saponins [24].

#### 2.3.3. Olefinic Protons and Carbons

Commonly found olefinic hydrogen H-6 and exomethylene protons H_2–_27 in spirostane analogues resonate at *δ* 5.25–5.60 and 4.75–4.85, respectively. Olefinic carbons resonate at *δ*_C_ 139–140 (C-5), 121–125 (C-6), 140–145 (C-25), and 108–110 (C-27) in spirostanes and at about *δ*_C_ 104 (C-20), 152-157 (C-22) in the case of furostane analogues.

#### 2.3.4. Sugar Units

The protons attached to oxygenated C-1, C-3 and C-16 resonate at *δ* 3.65–3.85 (dd, *J* ~12 and 4 Hz), 3.85–4.05 (m), and 4.45–4.56 (dd, *J* ~ 14.5 and 8 Hz), respectively. Most of the sugar protons resonate in a narrow range (*δ* 3.0–4.5) and are highly overlapped. Using high-field (≥400 MHz) NMR instruments the geminal protons of a C-6 oxygenated sugar are usually identifiable as AB part of a ABX system (H_a_-6: *δ* ~ 4.35–4.45, dd, *J* ~ 12 and 2 Hz; H_b_-6: *δ* ~ 4.30–4.35, dd, *J* ~ 12 and 5.5 Hz), whereas the H_2_-5 protons of an α-arabinopyranosyl unit give rise to two signals at *δ* ~ 3.65 (d, *J* ~ 11 Hz) and ~4.25 (m). The anomeric protons are also clearly distinguishable as doublets in the region of *δ* 4.1–6.4 with coupling constants (^3^*J*) between H-1 and H-2 in the range of ~7–9 Hz for a diaxial orientation and ~ 1–3 Hz, for an axial/ equatorial or diequatorial arrangement [40]. Therefore, the α/β configuration of the anomeric proton can be inferred from the value of the ^3^*J*_CH_ coupling that follows the Karplus relationship. For example, the H-1 of a β-glucopyranosyl unit usually resonates at *δ* 4.8–5.5 as a doublet with *J* ~ 7–8.5 Hz, whereas the doublet (often broad singlet) for the H-1 of an α-rhamnopyranosyl unit usually appears at *δ* 5.7–6.4 with *J* ~ 1.1–1.7 Hz, the signal for the H-1 of an α-arabinopyranosyl unit usually resonates as a doublet at *δ* 4.6–4.75 with *J* ~ 7 Hz, and the doublet of the anomeric proton of a β-xylopyranosyl unit appears at *δ* 5.10–5.75 with *J* ~ 6–8 Hz [40]. The α-configuration of an α-rhamnopyranosyl moiety is also supported by the axial/axial relationship between H-3/H-4 and H-4/H-5 (*J* > 9 Hz) and the axial-equatorial H-2/H-3 (*J* < 5 Hz).

In the ^13^C NMR spectra of saponins, the sugar anomeric carbons resonate in the chemical shift range of *δ*_C_ 96–112, allowing to infer the number of monosaccharide units present and sometimes also the nature of the glycosidic linkages. The C-22 quaternary acetal carbon also resonates at about *δ*_C_ 110. These signals are well differentiated from the other sugar oxygenated carbons that appear in the range of *δ*_C_ 60–90, with the carbon of a CH_2_OH group (e.g., C-6 of a glucopyranosyl moiety) resonating at about *δ*_C_ 62.5 and that of a CH_2_O group (e.g., C-5 of an arabinopyranosyl moiety) appearing at about *δ*_C_ 68. Oxygenated carbons of the sapogenin core occur in the same region as the oxygenated sugar carbons. It is noteworthy that glycosylation causes a downfield shift of 0.5 and 7–12 ppm for the proton and the carbon of a non-anomeric CHO group involved in a glycosidic linkage, respectively, and an upfield shift of 2–5 ppm for the β-carbon [41]. Therefore, the terminal monosaccharide does not show any glycosylation shift. These effects are, thus, useful for establishing the glycosylation pattern.

In addition to the information inferred from the 1D NMR spectra, a combination of 2D NMR techniques such as COSY, HOHAHA or TOCSY, HETCOR or HMQC, HMBC, NOESY or ROESY [42,43,44] are routinely used to establish or confirm the identity of the aglycone, the sugars, and the sugar sequence of the oligosaccharide substituents. Instead, INADEQUATE experiments can rarely be used because they are large sample demanding. Short structural fragments are identified by COSY or HETCOR/HSQC-TOCSY spectra; they are then bonded to each other using the information obtained from NOESY/ROESY and HMBC experiments. NOESY/ROESY spectra also help to establish the configuration of stereocenters. As regards the sugar residues, the spin systems from the anomeric to the terminal proton of each monomer are identified using several 1D-TOCSY and 1D-ROESY experiments with different mixing times [25]. The ^13^C sugar signals can then be assigned unambiguously with the help of a HETCOR or HMQC experiment. Finally, NOESY/ROESY and HMBC measurements allow to identify the sugar sequence and the inter-glycosidic bonds. In fact, the presence of an inter-glycosidic NOE from the anomeric proton of a sugar residue to a proton of another sugar or to a sapogenin proton suggests the existence of a glycosidic linkage between the two residues. For example, a NOE correlation between the anomeric proton of the first sugar and the H-3 of aglycone confirms the placement of the oligosaccharide chain at C-3. A ^3^*J*_CH_ coupling (HMBC) between the anomeric proton and the aglycone carbon or sugar carbon to which it is linked firmly confirms the glycosidic linkage. For example, in the case of furostanol glycosides, HMBC and ROESY cross peaks between the anomeric proton of a glucopyranosyl moiety and C-26 or H_2_-26 of the aglycone confirm the glycosylation of 26-OH.

Numerous informative examples of the application of these techniques can be found in publications reporting saponins listed in the following tables [26,27,28,29,30,31,32,33].

## 3. Steroidal Saponins Isolated from *Dracaena* and *Sansevieria* Species

**Figure 2:**
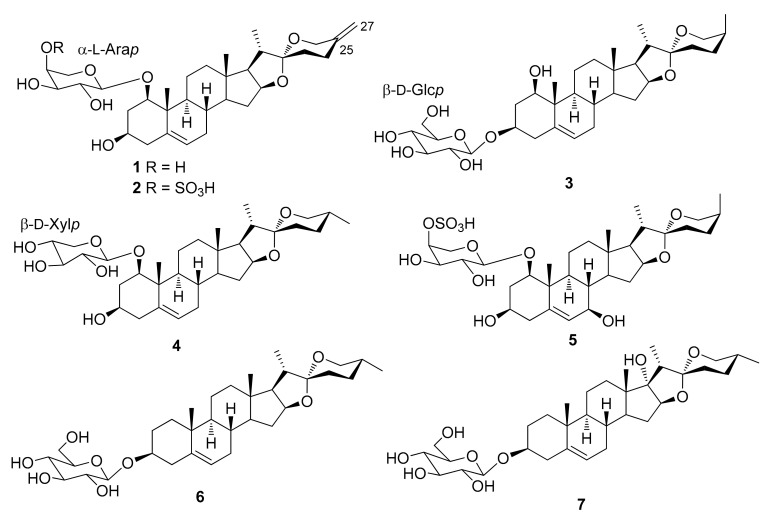
Chemical structures of spirostanol monoglycosides isolated from *Dracaena* and *Sansevieria* species.

**Figure 3:**
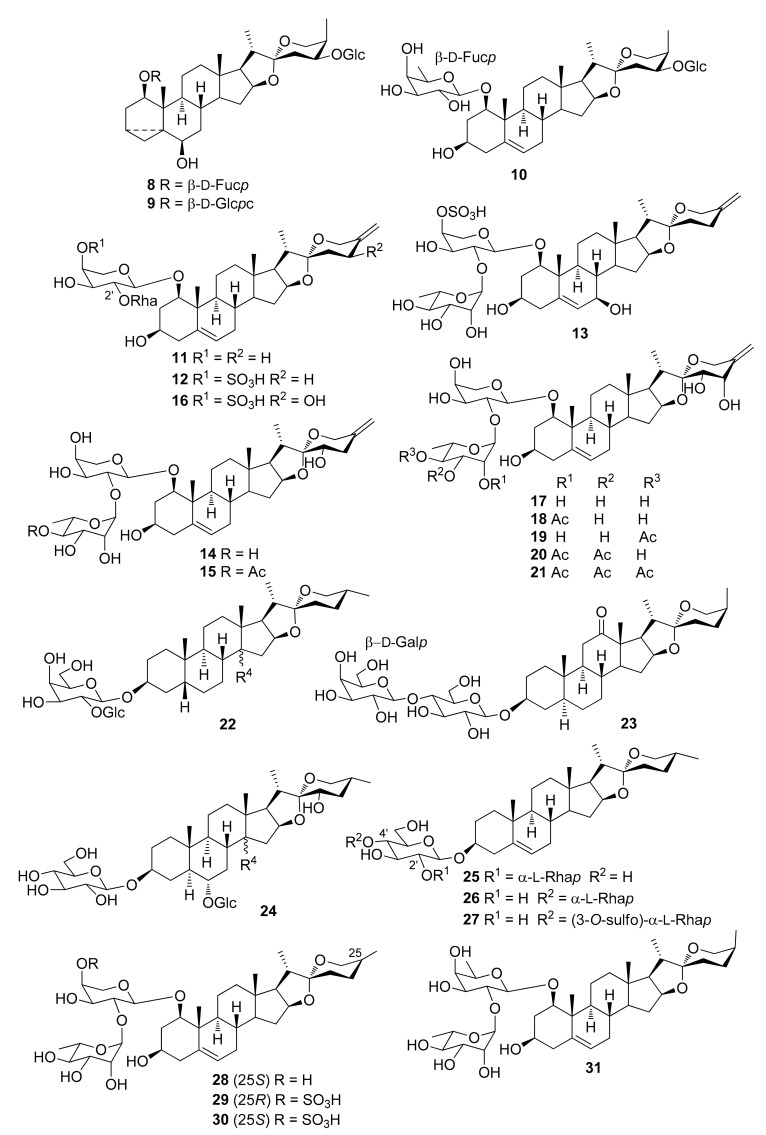

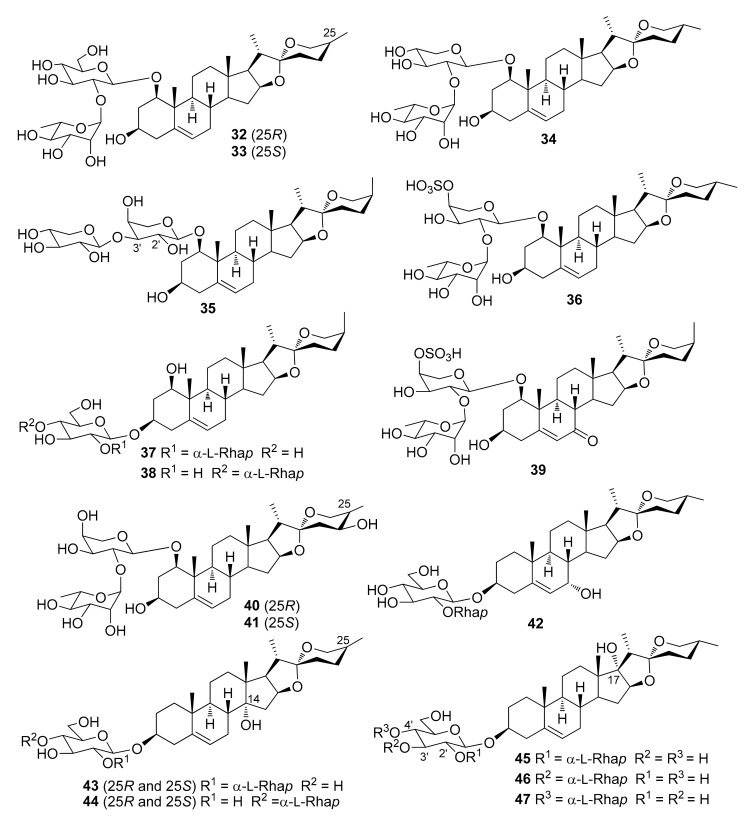
Chemical structures of spirostanol diglycosides isolated from *Dracaena* and *Sansevieria* species.

**Figure 4:**
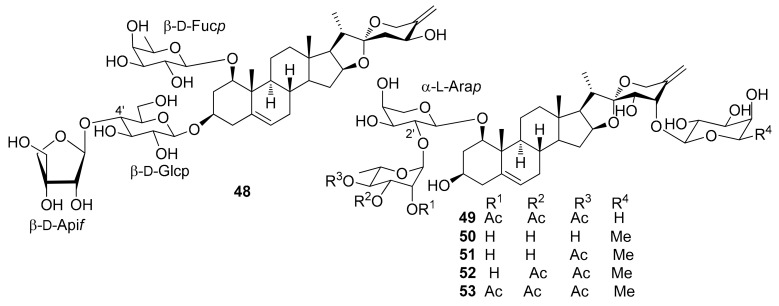

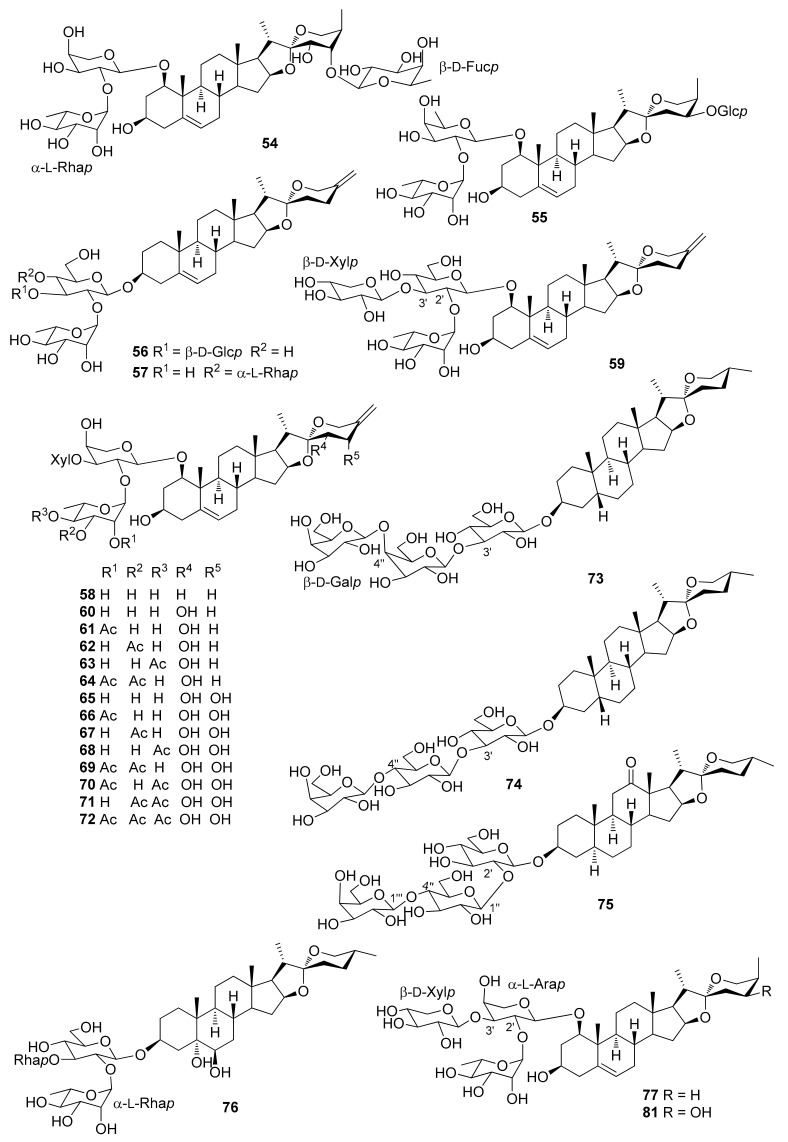

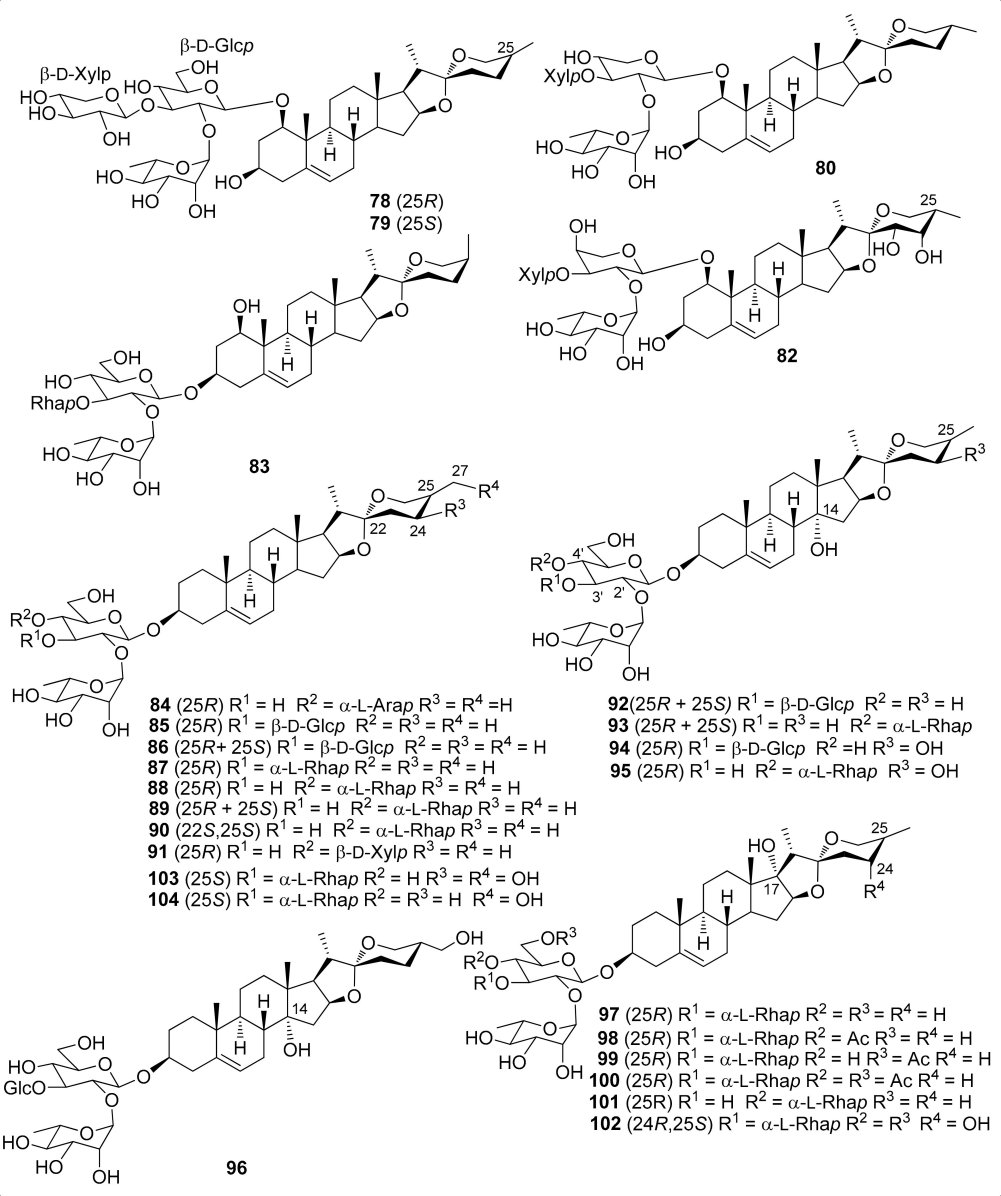
Chemical structures of spirostanol triglycosides isolated from *Dracaena* and *Sansevieria* species.

**Figure 5:**
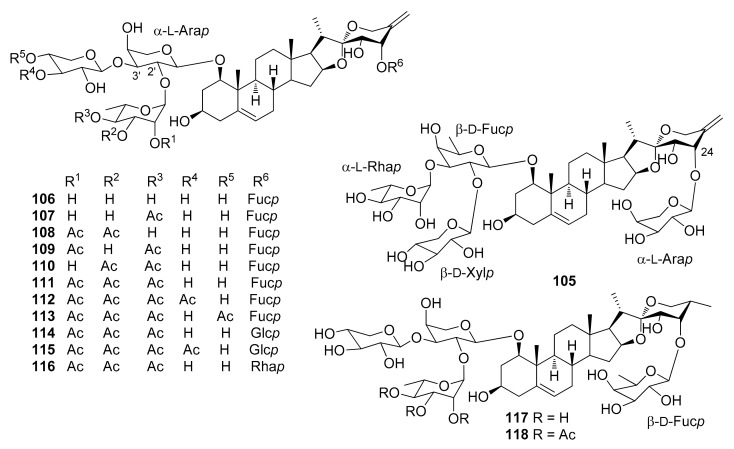
Chemical structures of spirostanol tetraglycosides isolated from *Dracaena* and *Sansevieria* species.

**Figure 6:**
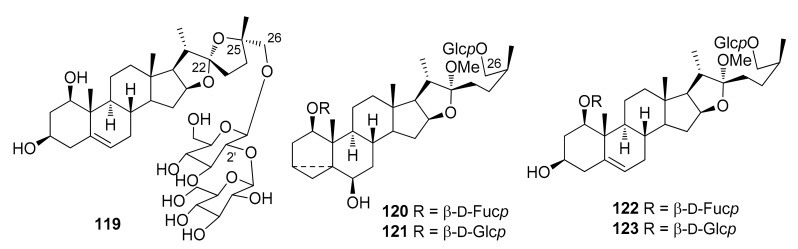
Chemical structures of furostanol diglycosides isolated from *Dracaena* species.

**Figure 7:**
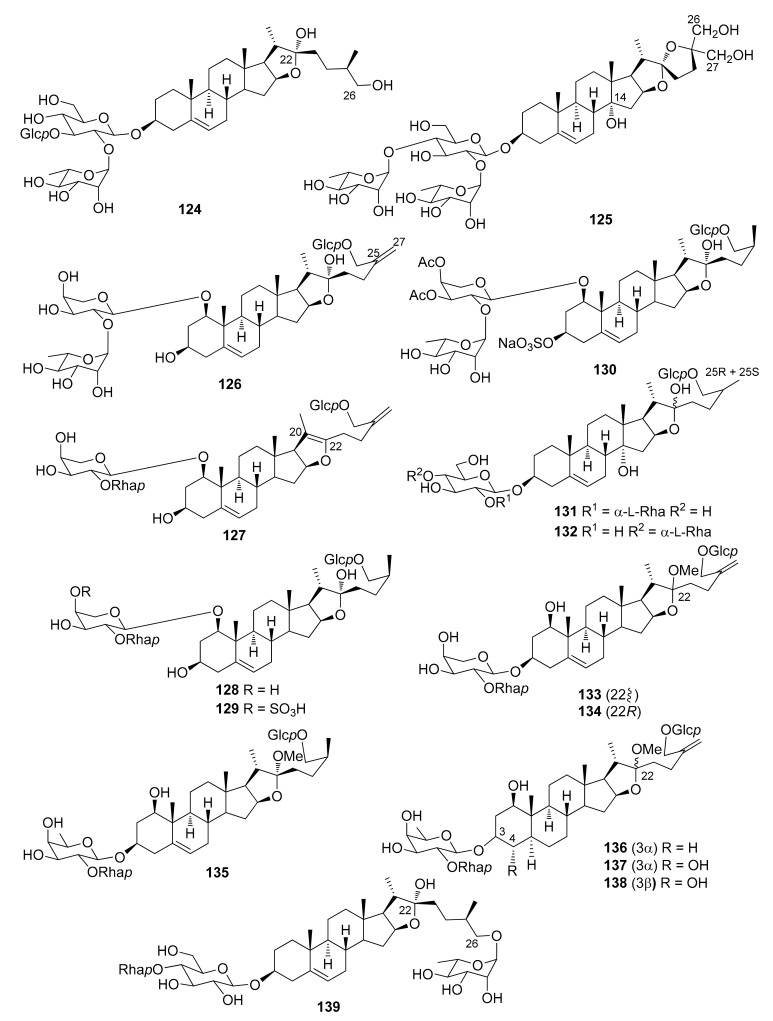
Chemical structures of furostanol triglycosides isolated from *Dracaena* and *Sansevieria* species.

**Figure 8:**
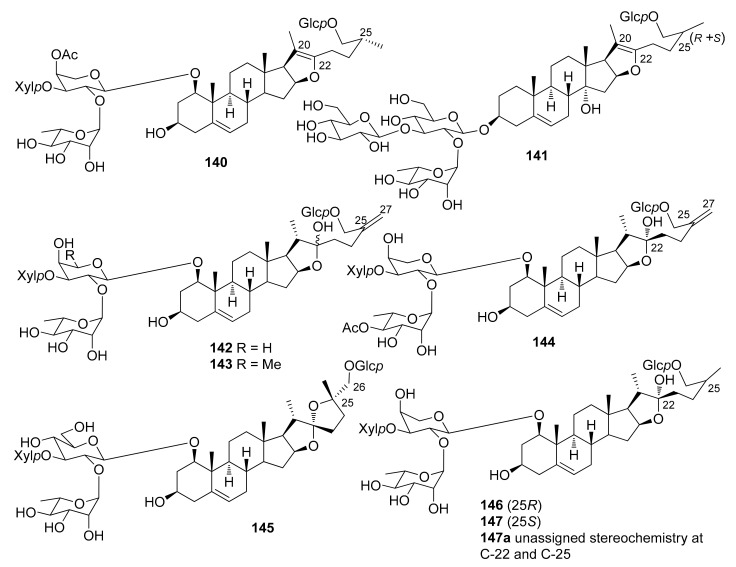

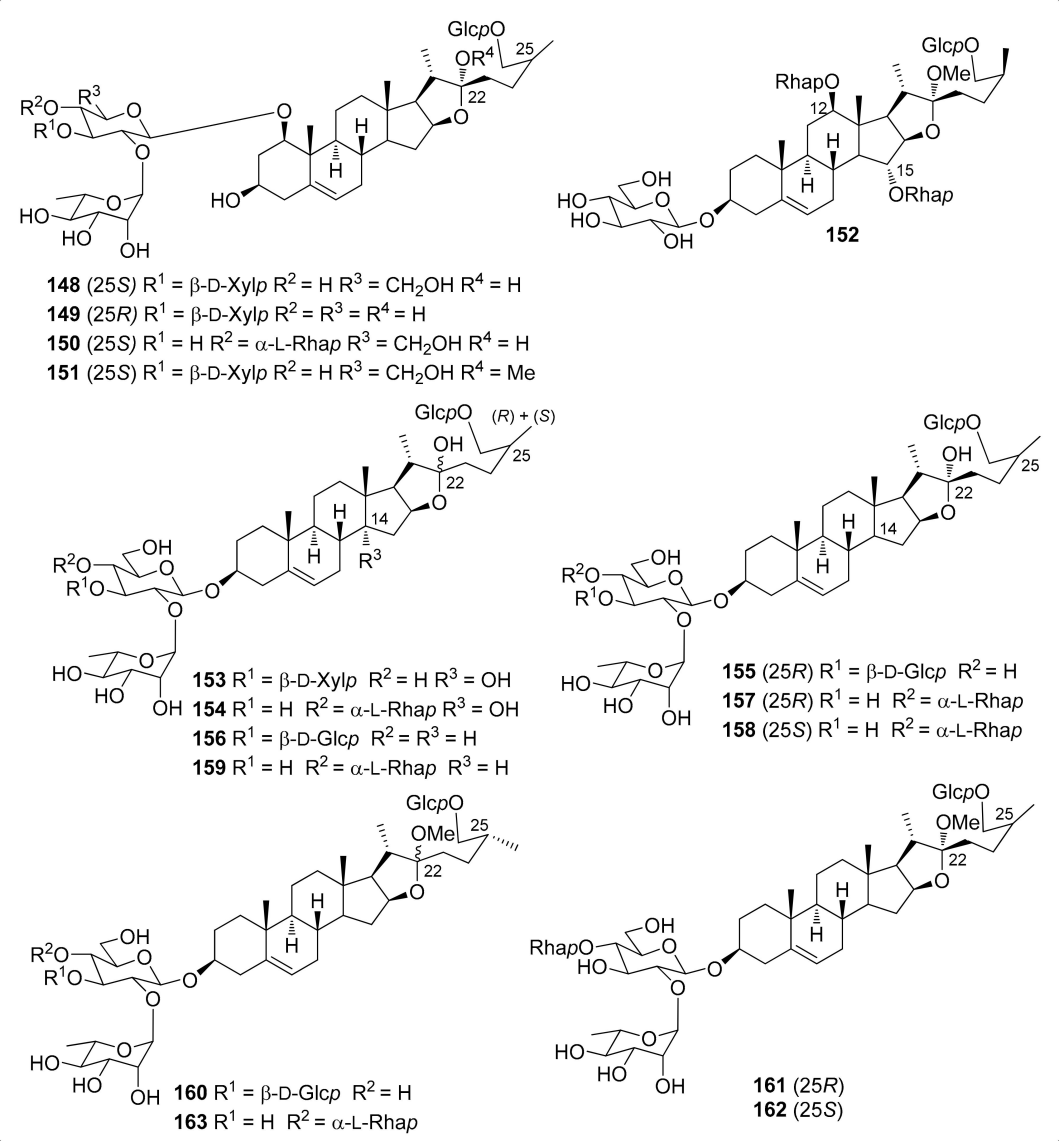
Chemical structures of furostanol tetraglycosides isolated from *Dracaena* and *Sansevieria* species.

**Figure 9:**
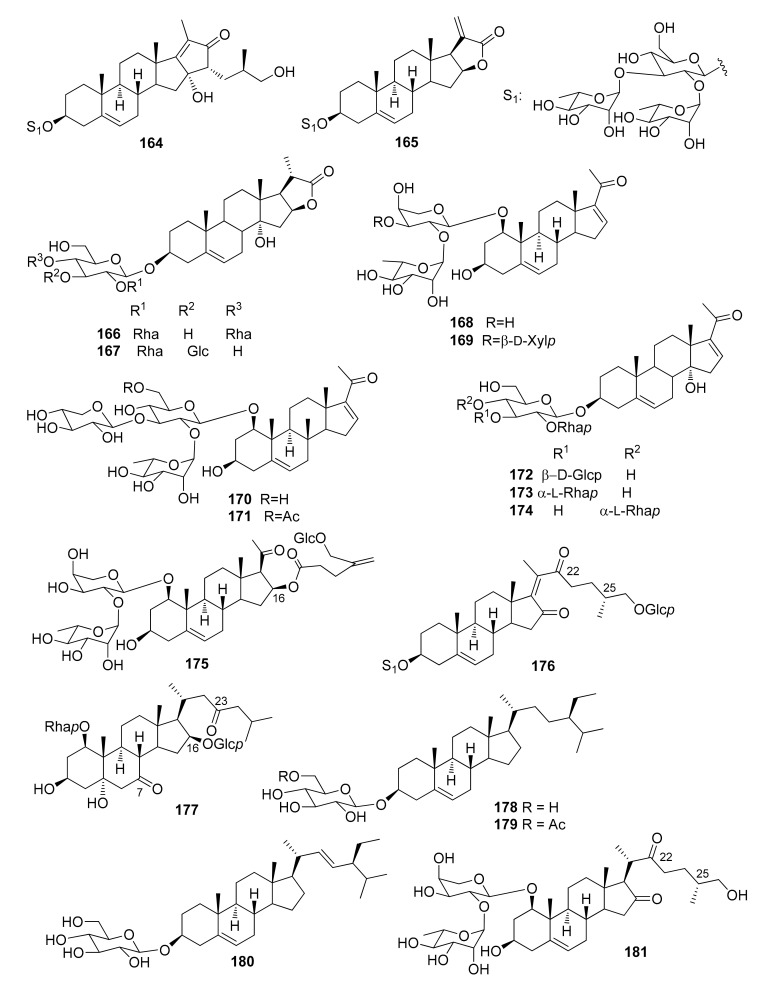
Chemical structures of miscellaneous steroidal saponins isolated from *Dracaena* and *Sansevieria* species.

The steroidal saponins isolated from *Dracaena* and *Sansevieria* species are listed in the following tables, in accordance with the number of sugar residues bonded to aglycone. They have been divided between spirostane (Table 2, Table 3, Table 4, Table 5), furostane (Table 6, Table 7, Table 8) and miscellaneous saponins (Table 9). The occurrence of saponins in *Dracaena* and *Sansevieria* species is shown in Table 10, whereas biological properties are reported in Table 11 and Table 12. The chemical structures are shown in Figure 2, Figure 3, Figure 4, Figure 5, Figure 6, Figure 7, Figure 8, Figure 9.

Table 10 shows the pattern of saponins in each *Dracaena* and *Sansevieria* species. It appears that each plant produces its own group of saponins. The richest species are *D. angustifolia*, *D. cambodiana*, *D. cochinchinensis*, *D. draco*, *D. thalioides*, and *S. trifasciata*. Saponins occurring in all species, that may be considered chemotaxonomic markers of the two genera, have not been isolated; saponins **11**, **25**, **28**, **58**, **77**, **88**, and **97** occur in more than three species.

## 4. Biological Activities

The spectrum of biological effects tested for the saponins that are isolated from *Dracaena* and *Sansevieria* species is rather limited, and in vitro assays have been performed in most cases. In this review, we have reported the biological activities determined during the isolation of individual saponins from these two genera. The bioactivities of extracts or saponin-enriched fractions have not been discussed. Additional data, collected during the isolation of the same saponins from other genera, have not been reported and for them readers should consult the original literature.

### 4.1. Hemolytic Properties

Most saponins have powerful haemolytic activities because steroids have high affinities for cholesterol on erythrocyte membranes, thereby altering membrane permeability. Therefore, their toxicity is high when saponins are given intravenously to higher animals, while it is much less when administered orally. In this context, it is worthy of note that compound **152** showed no haemolytic effects in vitro [69]. This special behaviour was explained by assuming that the distribution of the sugar units around the aglycone moiety of the saponin considerably reduces its hydrophobicity, resulting in the loss of the amphipathic features. Moreover, saponin **152** inhibited the increase in capillary permeability caused by acetic acid, which is a typical model of first stage inflammatory reaction [69].

### 4.2. Antiinflammatory Activity

Pennogenin glycosides mannioside A (**46**) and floribundasaponin A (**7**) significantly inhibited carrageenan-induced paw edema in the rat, whereas spiroconazole A (**97**) was moderately active and aglycone pennogenin showed very weak activity [51]. The highest anti-inflammatory effects were observed one hour after carrageenan injection (Table 11), whereas the reference drug indomethacin reached a maximum of inhibition (62.36%) at the 4th hour. These data underline the importance of the presence of a glycoside unit for the anti-inflammatory activity of pennogenin derivatives. The highest effects where observed when D-glucose was attached to 3-OH of pennogenin and a rhamnosyl moiety was attached to 3′-OH [51].

Neutrophils play a significant role in the pathogenesis of several inflammatory diseases. The production of vast amounts of superoxide anion and elastase by activated neutrophils can cause tissue damage and contribute to the development of a wide spectrum of airway inflammatory diseases [58]. In this context, drangustosides A (**83**) and B (**37**) exhibited inhibitory activities from moderate to high against formyl-L-methionyl-L-leucyl-L-phenylalanine (fMLP)-induced superoxide anion production and elastase release in human neutrophils [58].

### 4.3. Antimicrobial Activity

A few compounds were very active against the pathogenic yeasts *Cryptococcus neoformans* and *Candida albicans* (Table 11), with most minimum inhibitory concentrations (MICs) in the 1–2 µg/mL range. Authors demonstrated that the antifungal activity of saponin 91 was fungicidal [57].

**Table 11:** Miscellaneous bioactivities of saponins isolated from *Dracaena* and *Sansevieria* species.

Bioactivity	Saponin (Ref.)	Description
Haemolytic effects	**152** [69]	No haemolytic effects and inhibition of the capillary permeability activity
Anti-inflammatory activity	**7** [51]	Anti-inflammatory activity on carrageenan-induced paw edema (maximum inhibitory activity of 71.22%)
	**46** [51]	Anti-inflammatory activity on carrageenan-induced paw edema (maximum inhibitory activity of 80.57%)
	**97** [51]	Anti-inflammatory activity on carrageenan-induced paw edema (maximum inhibitory activity of 66.19%)
Anti-neutrophilic inflammatory activity	**37** [58]	Inhibitory activity against formyl-L-methionyl-L-leucyl-L-phenylalanine-induced superoxide anion generation (IC_50_ = 18.55 ± 0.23 μM) and elastase release by human neutrophils (IC_50_ = 1.74 ± 0.25 μM)
	**83** [58]	Inhibitory activity against formyl-L-methionyl-L-leucyl-L-phenylalanine-induced superoxide anion generation (IC_50_ = 26.39 ± 1.63 μM) and elastase release by human neutrophils (IC_50_ = 3.94 ± 0.19 μM)
Antimicrobial activity	**25** [57]	Antimicrobial activities against *Cryptococcus neoformans* (MIC = 1 μg/mL) and *Candida albicans* (MIC = 2 μg/mL)
	**28** [48]	Antifungal activity against *Cryptococcus neoformans* (IC_50_ = 20.0 μg/mL)
	**32** [62]	Antibacterial activity against *Staphylococcus aureus*
	**35** [48]	Antifungal activity against *Cryptococcus neoformans* (IC_50_ = 9.5 μg/mL)
	**57** [57]	Antimicrobial activities against *Cryptococcus neoformans* (MIC = 1–2 μg/mL) and *Candida albicans* (MIC = 2 μg/mL)
	**84** [57]	Antimicrobial activities against *Cryptococcus neoformans* (MIC = 1–2 μg/mL)
	**88** [57]	Antimicrobial activities against *Cryptococcus neoformans* (MIC = 1–2 μg/mL) and *Candida albicans* (MIC = 2 μg/mL)
	**91** [57]	Antimicrobial activities against *Cryptococcus neoformans* (MIC = 2 μg/mL) and *Candida albicans* (MIC = 4–8 μg/mL)
Molluscicidal activity	**97** [66]	At the concentration of 5–6 ppm, spiroconazole A caused 100% mortality of the snails *Bulinus globosus*, *B. forskalii*, *Biomphalaria* pfeifferi, *B. glabrata*, and *Lymnaea natalensis* within 24 h. Other two related (unidentified) saponins were lethal with LC_50_ values in the range of 10–25 ppm

### 4.4. Molluscicidal Activity

Some snails, especially of the genera *Bulinus*, *Biomphalaria* or *Oncomelania* are implicated in the transmission of schistosomiasis (bilharzia). The infection is caused by cercariae, liberated from the intermediate host snail, that penetrate the skin and mainly affect the genitourinary and gastro-intestinal systems. Three spirostanol saponins, pennogenin glycosides, isolated from the methanolic extract of the fruit pulp of *Dracaena mannii*, exhibited significant molluscicidal activity when tested against 5 snail vectors [66]. The highest activity was shown by spiroconazole A (**97**) whose LC_50_ values were 2.95 ppm (*Bulinus globosus*), 4.36 ppm (*B. forskalii*) and 3.63 ppm (*Lymnaea natalensis*), respectively [66]. The aglycones showed no molluscicidal activity, indicating that the sugar moiety is an essential part of the molecules to retain the activity.

### 4.5. Cell Antiproliferative/Cytotoxic Activity

A relatively vast number of saponins, isolated from *Dracaena* and *Sansevieria* species, have been tested for their antiproliferative/cytotoxic activities in vitro (Table 12). However, the data are hardly comparable due to the fact that different cell lines and procedures were often used in different laboratories. The choice of cell lines (usually from one to five) in most reports, cited in this review, seemed to be random. It is, thus, highly recommendable that a standardized procedure is followed in future investigations. Moreover, antiproliferative activity and cytotoxicity have often been considered as synonyms and the terms, antiproliferation (cytostatic effects) and cytotoxicity (killing effects), have been used interchangeably. Most assays have been performed with a colorimetric method (MTT, XTT), that is based on the reduction of tetrazolium salts in living cells by mitochondrial dehydrogenases. This assay allows, indeed, the estimation of the activity of metabolically active cells (viable cells), while offering little insight into the mechanisms concerning antiproliferative versus cytotoxic effects. The sulforhodamine B (SRB) assay has also been used to determine cell growth inhibition [49]. DNA damage and the consequent induction of apoptosis are principal cytotoxic mechanisms of several anticancer agents. Therefore, in certain cytotoxicity assays DNA fragmentation, condensation of chromatin and other morphological changes are visualized and quantified by fluorescence microscopy. Immunoblot (Western blot) analysis is also performed to determine cleavage of poly(ADP-ribose) polymerase-1 (PARP), which it is a key enzyme in the apoptosis process. Moreover, the cytotoxic effects of some steroidal saponins may result from their nonspecific detergent effects on membrane architecture [57], even if there is no clear correlation between the haemolytic activity and cytotoxicity of steroidal saponins [73].

We have maintained the distinction between cytostatic and cytotoxic activities. Table 12 show the cell growth inhibition activities of saponins as IC_50_ values, i.e., the concentration at which 50% inhibition of cell growth was observed. For homogeneity, the values expressed in literature in µg/mL have been converted to µM. Human tumour cell lines were used where not otherwise indicated. IC_50_ values up to 30 µM have been included in the table, even if several authors consider the activity of a compound worthy of note only if the IC_50_ is <10 µM. Indeed, a great number of saponins from *Dracaena* and *Sansevieria* exhibited IC_50_ values in the range of 1–10 µM, and some in the submicromolar range. In addition, several saponins exhibited higher activity than the reference compound (e.g., etoposide, paclitaxel, doxorubicin, 5-fluorouracil) on certain cell lines.

A rather comprehensive review on saponin cytotoxicity was published a few years ago [73]. The structure-activity relationships of saponins are generally difficult to decipher, and only a fairly limited number of comparative studies have been focused on the establishment of structure-activity relationships, relevant to activity [73]. From the data reported in Table 12, and others in the literature, it appears that the a glycoside unit is essential for cytostatic/cytotoxic activities of steroidal saponins. Moreover, the activity is sensitive to the monosaccharides constituting the sugar moieties, and their number and sequences, the glycosylation site, as well as the structures of the aglycones. For example, the 25*S* configuration appeared to be critical for activity against leukemia cells but not against solid tumors [73]. Furthermore, the presence of oxygenated functional groups on some positions of the aglycones, e.g., at C-7, seems to reduce the activity. The 1-*O* glycosylated saponins **111**, **112** and **118** were the most active among a number of saponins tested against promyelocytic leukemia HL-60 cells; they showed IC_50_ values comparable or even higher than the well-known chemotherapeutic agents cisplatin and etoposide (Table 12). The authors suggested that the triacetylated α-L-rhamnopyranosyl moiety at C-2′ and the β-D-fucopyranosyl group attached to C-24 of the aglycone play important roles in imparting potent antiproliferative activity against HL-60 cells [32]. However, dioscin (**88**) bearing a non-acylated Rhap-(1→2)-[Rhap-(1→4)]-Glcp moiety at C-3 of the aglycone, was also quite active against HL-60 cells [28], while fully acetylated dioscin was inactive against several cell lines, contrary to dioscin [49]. In the dioscin series, comparison of the activity of 2′,4′-di-*O*-rhamnopyranosyl-3-*O*-glucopyranoside **88** with those of monorhamnosyl derivatives **25** and **26** (Table 12), and inactive monoglycosylated trillin **6** [50], clearly shows that the antiproliferative activity is greatly increased in the dirhamnosyl derivative, while the presence of a rhamnosyl unit at C-2′ is critical to the activity. Substitution of the 4′-rhamnosyl unit in dioscin with an arabinopyranosyl or a xylopyranosyl moiety, slightly modifies the activity (cfr. **88** with **84** and **91**, Table 12). Icodeside (**49**) and draconin A (**21**), exhibited similar antiproliferative activity towards HL-60 cells (IC_50_ ca. 9) [28,29], which indicates that the additional arabinopyranosyl moiety in **49**, compared to draconin A, does not affect the activity significantly. Considering the antiproliferative activity of the saponins isolated from the roots and rhizomes of *D. angustifolia* [26], the authors concluded that the spirostanol saponins possess more potent antiproliferative activity than their furostanol analogues. In contrast, furostanol icogenin (**88**) is even slightly more active than the correspondin spirostanol gracillin (**85**) against HL-60 cells [55]. A 24-*O*-fucopyranosyl substituent, together with a triacetylated α-L-rhamnopyranosyl moiety and a xylopranosyl unit attached to the 2′-*O* and 3′-*O*, respectively, of an arabinopyranosyl substituent at 1-*O* seem to be important for the activity against HT-1080 fibrosarcoma cells (Table 12).

In addition to antiproliferative activities, saponins **20**, **21**, **85**, **112**, **124** also exhibited significantly cytotoxic properties [28,32,55] and some mechanisms of action have been clarified. For example, the potent anticancer agent gracillin (**85**) showed significant inhibitory effect on mitochondrial complex II-mediated energy production in non-small cell lung cancer, and it inhibited glycolysis and oxidative phosphorylation-mediated bioenergetics [74].

**Table 12:** Antiproliferative activities of saponins isolated from *Dracaena* and *Sansevieria* species.

Drug (Ref.)	Cell Line IC_50_ (µM)								
	Promye- locytic leukemia HL-60								
cisplatin [32]	1.40 ± 0.08								
etoposide [32]	0.38 ± 0.06								
**1** [32]	9.34 ± 2.93								
**4** [32]	7.38 ± 0.78								
**11** [32]	7.85 ± 0.43								
**32** [32]	17.3 ± 2.99								
**34** [32]	12.3 ± 2.56								
**46** [32]	20								
**59** [32]	9.45 ± 2.22								
**73** [32]	4.45 ± 0.39								
**80** [32]	11.3 ± 1.21								
**97** [32]	6.36 ± 0.14								
**98** [32]	7.64 ± 0.59								
**100**[32]	>20								
**108** [32]	6.00 ± 1.22								
**111** [32]	0.47 ± 0.04								
**112** [32]	0.38 ± 0.04								
**114** [32]	2.73 ± 0.42								
**115** [32]	1.66 ± 0.20								
**118** [32]	0.74 ± 0.05								
**21** [28]	9.7 ± 2.7								
**85** [28]	3.7 ± 0								
**85** [55]	4.0 ± 0.4								
**88** [28]	2.0 ± 0.9								
**88** [55]	2.3 ± 0.8								
**155** [28]	7.2 ± 2.3								
**157** [28]	7.3 ± 3.7								
**49** [29]	9 ± 4								
etoposide [29]	0.2								
**25** [53]	1.8								
**53** [53]	2.5								
etoposide [53]	0.5								
**124** [55]	2.6 ± 0.9	Epi-dermoid carcinoma A-431	HeLa (derived from cervical cancer cells)	Colo rectal cancer HCT116	Hepato cyte carcinoma HepG2	Breast carci noma MCF7	Myelo genous leuke mia K-562	Hepatoma BEL-7402	Gastric cancer SGC-7901
**49** [29]		16.1							
**109** [33]			26.5						
**110** [33]			26.5						
**33** [47]						29.6 ± 1.4			
**38** [47]				16.9 ± 1.4	15.5 ± 2.8	18.3 ± 1.4			
**77** [47]				8.3 ± 2.3	10.7 ± 2.3	4.8 ± 2.3			
**146** [47]				24.5 ± 1	18.6 ± 1	20.6 ± 1			
doxorubicin HCl [47]				22.4 ± 1.7	3.4 ± 5.1	1.7 ± 1.7			
**58** [45]							4.77	6.44	5.61
**87** [45]							1.27	4.72	2.88
**97** [45]							5.09	1.13	3.39
paclitaxel [45]							5.98	3.75	1.88
	Fibro-sarcoma HT-1080	Murine colon carcinoma 26-L5	MelanomaB16-BL6						
**17** [26]		5.3	4.2						
**50** [26]	27.7								
**51** [26]	21.6								
**53** [26]	3.8	30.2	20.9						
**72** [26]	11.1		28.4						
**111** [26]	0.6	22.1	11.9						
**112** [26]	0.2	26.6	9.7						
**113** [26]	0.3	27.7	11.8						
**140** [26]	21.8								
5-fluoro uracil [26]	1.5	0.5	0.6						
doxorubicin HCl [26]	0.2	0.1	0.2	Lympho cytic leukemia P388	Pancreas Carci noma BXPC-3		CNS glioblastoma SF268	Lung NCI-H460	Colon carcinoma KM20L2
**25** [57]				2.1	2.5	2.8	2.5	2.5	2.4
**57** [57]				1.8	1.3	1.3	1.5	0.5	0.5
**84** [57]				2.0	1.1	0.7	0.8	0.3	0.3
**88** [57]				1.7	1.3	1.8	1.4	1.8	1.8
**91** [57]				3.0	2.0	1.6	1.5	1.4	0.6
	Prostate carcinoma DU-145	Lung carcinomaA549	T-cell leukemia Jurkat	Ovarian cancer Skov-3	Epithelial colorectal adenocarcinoma CaCo-2	Colon cancer SW480	Mouse mam mary cancer EMT6		
**25** [57]	2. 2								
**57** [57]	1.1								
**84** [57]	0.5								
**88** [57]	1.8								
**91** [57]	1.3								
**6** [50]		24.51 ± 0.17							
**25** [50]		2.91 ± 0.75	2.85 ± 0.16	7.87 ± 0.12	3.47 ± 0.44				
**26** [50]			30.07 ± 2.49		18.98 ± 1.16				
**88** [50]		0.48 ± 0.17	1.96 ± 0.44	2.19 ± 0.99	2.97 ± 0.24				
**161** [50]		4.94 ± 0.27	4.53 ± 0.31	6.6 ± 0.4	15.2 ± 0.3				
doxorubicin [49,50]		2,1 ± 1.5	0.1 ± 0.07	1.5 ± 0.15	4.3 ± 1.9	1.47	9.21		
**158** [49]						14.3	8.6		

## 5. Conclusions

We believe that this review clearly demonstrates that *Dracaena* and *Sansevieria* species are rich sources of steroidal saponins, and have intriguing structures and interesting biological properties, including high cell antiproliferative/cytotoxic and anti-inflammatory activities. Less than 10% of the total number of known species have been subjected to scientific studies so far and more than 75% of isolated saponins were new. These data indicate that the study of *Dracaena* and *Sansevieria* saponins is still a promising field of research, highlighting the importance of extending phytochemical investigations to more species, especially in search of novel bioactive constituents. Classical methods, such as Soxhlet extraction and maceration have been used so far for the extraction of *Dracaena* and *Sansevieria* saponins. One expects that less time-consuming and greener methods, such as ultra-sound assisted extraction, microwave-assisted extraction, super-critical fluid extraction and accelerated solvent extraction that usually increase the overall efficiency of the extraction procedures, will be used more frequently in future studies. Moreover, modern dereplication methods of analysis, such as LC-MS and other hyphenated techniques [75], may allow a straightforward way to propose the tentative identification of compounds [76].

Several bioactive saponins from *Dracaena* and *Sansevieria* have the potential to become lead compounds for the development of anticancer therapeutic agents. Further studies are required to understand the relevance and selectivity of the cytotoxic activity, and to define the mechanisms of action. It is also important to access potential risks and side effects. Moreover, most of the reports were in vitro studies; therefore, further animal experiments are essential to evaluate saponin activities.

## Figures and Tables

**Table 1:** Glycosylation patterns of *Dracaena* and *Sansevieria* saponins. ^a,b^

	1-OH	3-	6-	12-	15-	16-	24-	26-	2′-	3′-	4′-	4″-
Spirostanol monoglycosides	Ara*p* 3 Xyl*p* 1	Glc*p* 3	--	--	--	--	--	--	--	--	--	--
Spirostanol diglycosides	Ara*p* 19 Glc*p* 3 Fuc*p* 3 Xyl*p* 1	Gal*p*1 Glc*p* 15	Glc*p* 1	--	--	--	Glc*p* 3	--	Glc*p* 1 Rha*p* 28	Rha*p* 1 Xyl*p* 1	Gal*p* 1 Rha*p* 6	--
Spirostanol triglycosides	Ara*p* 23 Fuc*p* 2 Glc*p* 3 Xyl*p* 1	Glc*p* 32	--	--	--	--	Ara*p* 1 Fuc*p* 5 Glc*p* 1	--	Rha*p* 56 Glc*p* 1	Gal*p* 1 Glc*p* 8 Rha*p* 9 Xyl*p* 21	Api*f* 1 Ara*p* 1 Rha*p* 7 Xyl*p* 1	Gal*p* 3
Spirostanol tetraglycosides	Ara*p* 13 Fuc*p* 1	--	--	--	--	--	Ara*p* 1 Fuc*p* 10 Glc*p* 2 Rha*p* 1	--	Rha*p* 13 Xyl*p* 1	Rha*p* 1 Xyl*p* 13	--	--
Furostanol diglycosides	Fuc*p* 2 Glc*p* 2	--	--	--	--	--	--	Glc*p* 5	Glc*p* 1	--	--	--
Furostanol triglycosides	Ara*p* 5	Ara*p* 2 Fuc*p* 4 Glc*p* 7	--	--	--	--	--	Glc*p* 15 Rha*p* 1	Rha*p* 15	Glc*p* 1	Rha*p* 4	--
Furostanol tetraglycosides	Ara*p* 6 Fuc*p* 1 Glc*p* 4 Xyl*p* 1	Glc*p* 18	--	Rhap 1	Rha*p* 1	--	--	Glc*p* 30	Rha*p* 29	Glc*p* 6 Xyl*p* 13	Rha*p* 10	--
Cholestane derivatives	Ara*p* 1 Rhap 1	Glc*p* 4				Glc*p* 1		Glc*p* 1	Rha*p* 3	Rha*p* 2	--	--
Pregnanes and lactones **165**-**167**	Ara*p* 3 Glc*p* 2	Glc*p* 6						Glc*p* 1	Rha*p* 11	Glc*p* 2 Rha*p* 2 Xyl*p* 3	Rha*p* 2	

^a^ The number near each sugar code indicates the number of saponins containing that sugar bonded to the indicated OH. ^b^ For the calculation, each stereoisomer in a mixture has been considered separately.

**Table 2:** Spirostanol monoglycosides isolated from *Dracaena* and *Sansevieria* species.

Number	Compound Name	Plant	References
**1**	(22*R*)-Spirosta-5,25(27)-diene-1β,3β-diol (neoruscogenin) 1-*O*-α-L-arabinopyranoside	*D. angustifolia*	[26]
		*D. fragrans* *(D. deisteliana)*	[27]
		*D. thalioides*	[32]
**2**	(22*R*)-Spirosta-5,25(27)-diene-1β,3β-diol (neoruscogenin) 1-*O*-(4-*O*-sulfo)-α-L-arabinopyranoside (cambodianoside F)	*D. cambodiana*	[45]
		*D. fragrans* *(D. deisteliana)*	[46]
**3**	(22*R*,25*S*)-Spirost-5-ene-1β,3β-diol [(*S*)-ruscogenin] 3-*O*-β-D-glucopyranoside	*S. cylindrica*	[47]
**4**	(22*R*,25*R*)-Spirost-5-ene-1β,3β-diol [(*R*)-ruscogenin] 1-*O*-β-D-xylopyranoside	*D. thalioides*	[32]
**5**	(22*R*,25*S*)-Spirost-5-ene-1β,3β,7β-triol 1-*O*-(4-*O*-sulfo)-α-L-arabinopyranoside (angudracanoside E)	*D. angustifolia*	[48]
**6**	(22*R*,25*R*)-Spirost-5-en-3β-ol 3-*O*-β-D-glucopyranoside (trillin)	*D. marginata* *D. viridiflora*	[49] 50]
**7**	(22*R*,25*R*)-Spirost-5-ene-3β,17α-diol 3-*O*-β-D-glucopyranoside (pennogenin 3-*O*-β-D-glucopyranoside or floribundasaponin A)	*D. arborea* *D. draco* *D. mannii*	[27] [29] [51]

**Table 3:** Spirostanol diglycosides isolated from *Dracaena* and *Sansevieria* species.

Number	Compound Name	Plant	References
**8**	(24*S*,25*R*)-24-*O*-β-D-Glucopyranosyl-3α,5α-cyclospirostane-1β,6β,24-triol 1-*O*-β-D-fucopyranoside	*D. sarculosa*	[52]
**9**	(24*S*,25*R*)-24-*O*-β-D-Glucopyranosyl-3α,5α-cyclospirostane-1β,6β,24-triol 1-*O*-β-D-glucopyranoside	*D. sarculosa*	[52]
**10**	(24*S*,25*R*)-24-*O*-β-D-Glucopyranosyl-spirost-5-ene-1β,3β,24-triol 1-*O*-β-D-fucopyranoside (surculoside B)	*D. sarculosa*	[31]
**11**	(22*R*)-Spirosta-5,25(27)-diene-1β,3β-diol (neoruscogenin) 1-*O*-α-L-rhamnopyranosyl-(1→2)-α-L-arabinopyranoside	*D. angustifolia*	[26]
		*D. cambodiana*	[45]
		*D. draco*	[29,53]
		*D. fragrans* (*D. deisteliana*)	[27]
		*D. thalioides*	[32]
		*S. trifasciata* (*D. trifasciata*)	[54]
**12**	(22*R*)-Spirosta-5,25(27)-diene-1β,3β-diol (neoruscogenin) 1-*O*-α-L-rhamnopyranosyl-(1→2)-4-*O*-sulfo-α-L-arabinopyranoside (angudracanoside B	*D. angustifolia*	[48]
		*D. cambodiana*	[45]
		*D. fragrans (D. deisteliana*)	[46]
**13**	(22*R*)-Spirosta-5,25(27)-diene-1β,3β,7β-triol 1-*O*-α-L-rhamnopyranosyl-(1→2)-4-*O*-sulfo-α-L-arabinopyranoside (angudracanoside C)	*D. angustifolia*	[48]
**14**	(22*S*,23*S*)-Spirosta-5,25(27)-diene-1β,3β,23-triol 1-*O*-α-L-rhamnopyranosyl-(1→2)-α-L-arabinopyranoside	*D. draco*	[53]
**15**	(22*S*,23*S*)-Spirosta-5,25(27)-diene-1β,3β,23-triol 1-*O*-(4-*O*-acetyl)-α-L-rhamnopyranosyl-(1→2)-α-L-arabinopyranoside	*D. draco*	[29,53]
**16**	(22*R,*24*S*)-Spirosta-5,25(27)-diene-1β,3β,24-triol 1-*O*-α-L-rhamnopyranosyl-(1→2)-4-*O*-sulfo-α-L-arabinopyranoside (angudracanoside D)	*D. angustifolia*	[48]
**17**	(22*S*,23*S*,24*S*)-Spirosta-5,25(27)-diene-1β,3β,23,24-tetraol 1-*O*-α-L-rhamnopyranosyl-(1→2)-*O*-α-L-arabinopyranoside (draconin B)	*D. angustifolia*	[26]
		*D. draco*	[28,29,53,55]
**18**	(22*S*,23*S*,24*S*)-Spirosta-5,25(27)-diene-1β,3β,23,24-tetraol 1-*O*-α-L-(2-*O*-acetyl)-rhamnopyranosyl-(1→2)-α-L-arabinopyranoside (draconin C)	*D. draco*	[28,29]
**19**	(22*S*,23*S*,24*S*)-Spirosta-5,25(27)-diene-1β,3β,23,24-tetraol 1-*O*-α-L-(4-*O*-acetyl)-rhamnopyranosyl-(1→2)-α-L-arabinopyranoside (draconin C)	*D. draco*	[28,29,53]
**20**	(22*S*,23*S*,24*S*)-Spirosta-5,25(27)-diene-1β,3β,23,24-tetraol 1-*O*-α-L-(2,3-di-*O*-acetyl)-rhamnopyranosyl-(1→2)-α-L-arabinopyranoside (draconin B)	*D. draco*	[28,29]
**21**	(22*S*,23*S*,24*S*)-Spirosta-5,25(27)-diene-1β,3β,23,24-tetraol 1-*O*-(2,3,4-tri-*O*-acetyl)-α-L-rhamnopyranosyl-(1→2)-α-L-arabinopyranoside (draconin A)	*D. draco*	[28,29]
**22**	(22*R*,25*R*)-5β-Spirostan-3-β-ol (smilagenin) 3-*O*-β-D-glucopyranosyl-(1→2)-β-D-galactopyranoside	*D. ombet*	[30]
**23**	(22*R*,25*S*)-5α-Spirostan-3-β-ol-12-one 3-*O*-β-D-galactopyranosyl-(1→4)-β-D-glucopyranoside (terreside B) ^a^	*D. angustifolia*	[48]
**24**	(2 2*S*,23*S*,25*R*)-5α-Spirostane-3β,6α,23-triol-3,6-di-*O*-β-D-glucopyranoside (cantalasaponin-1)	*D. cambodiana*	[56]
**25**	(22*R*,25*R*)-Spirost-5-en-3β-ol (diosgenin) 3-*O*-α-L-rhamnopyranosyl-(1→2)-β-D-glucopyranoside (prosapogenin A)	*D. draco*	[53]
		*D. fragrans* *(D. deisteliana)*	[46]
		*D. viridiflora*	[49]
		*S. ehrenbergii*	[57]
**26**	(22*R*,25*R*)-Spirost-5-en-3β-ol (diosgenin) 3-*O*-α-L-rhamnopyranosyl-(1→4)-β-D-glucopyranoside (prosapogenin B)	*D. draco* *D. viridiflora*	[28,55] [50]
**27**	(22*R*,25*R*)-Spirost-5-en-3β-ol 3-*O*-(3-*O*-sulfo)-α-L-rhamnosyl-(1→4)-β-D-glucopyranoside (deistelianoside A)	*D. fragrans* *(D. deisteliana)*	[27]
**28**	(22*R*,25*S*)-Spirost-5-ene-1β,3β-diol [(*R*)-ruscogenin] 1-*O*-α-L-rhamnopyranosyl-(1→2)-α-L-arabinopyranoside (alliospiroside A)	*D. angustifolia* *D. concinna* *D. marginata* *S. cylindrica*	[48,58] [59] [49] [60]
**29**	(22*R*,25*R*)-Spirost-5-ene-1β,3β-diol [(*R*)-ruscogenin] 1-*O*-α-L-rhamnopyranosyl-(1→2)-4-*O*-sulfo-α-L-arabinopyranoside	*D. angustifolia*	[48]
		*D. fragrans* *(D. deisteliana)*	[46]
**30**	(22*R*,25*S*)-Spirost-5-ene-1β,3β-diol [(*S*)-ruscogenin] 1-*O*-α-L-rhamnopyranosyl-(1→2)-4*-O-*sulfo-α-L-arabinopyranoside	*D. marginata*	[49]
**31**	(22*R*,25*S*)-Spirost-5-ene-1β,3β-diol [(*S*)-ruscogenin] 1-*O*-α-L-rhamnopyranosyl-(1→2)-β-D-fucopyranoside	*D. surculosa*	[31]
**32**	(22*R*,25*R*)-Spirost-5-ene-1β,3β-diol [(*R*)-ruscogenin] 1-*O*-α-L-rhamnopyranosyl-(1→2)-β-D-glucopyranoside	*D. marginata* *D. thalioides*	[49] [32]
**33**	(22*R*,25*S*)-Spirost-5-ene-1β,3β-diol [(*S*)-ruscogenin] 1-*O*-α-L-rhamnopyranosyl-(1→2)-β-D-glucopyranoside	*S. cylindrica*	[47]
**34**	(22*R*,25*R*)-Spirost-5-ene-1β,3β-diol [(*R*)-ruscogenin] 1-*O*-α-L-rhamnopyranosyl-(1→2)-β-D-xilopyranoside	*D. thalioides*	[32]
**35**	(22*R*,25*S*)-Spirost-5-ene-1β,3β-diol [(*S*)-ruscogenin] 1-*O*-β-D-xylopyranosyl-(1→3)-α-L-arabinopyranoside (angudracanoside F)	*D. angustifolia*	[48]
**36**	(22*R*,25*R*)-Spirost-5-ene-1β,3β-diol [(*R*)-ruscogenin] 3-*O*-α-L-rhamnopyranosyl-(1→2)-4*-O-*sulfo-α-L-arabinopyranoside	*D. concinna*	[59]
**37**	(22*R*,25*S*)-Spirost-5-ene-1β,3β-diol [(*S*)-ruscogenin] 3-*O*-α-L-rhamnopyranosyl-(1→2)-β-D-glucopyranoside (drangustoside B)	*D. angustifolia*	[58]
**38**	(22*R*,25*S*)-Spirost-5-ene-1β,3β-diol [(*S*)-ruscogenin] 3-*O*-α-L-rhamnopyranosyl-(1→4)-β-D-glucopyranoside	*S. cylindrica*	[47]
**39**	(22*R*,25*S*)-Spirost-5-ene-1β,3β-diol-7-one 1-*O*-α-L-rhamnopyranosyl-(1→2)-4-*O*-sulfo-α-L-arabinopyranoside (angudracanoside A)	*D. angustifolia*	[48]
**40**	(22*R*,24*S*,25*R*)-Spirost-5-ene-1β,3β,24-triol 1-*O*-α-L-rhamnopyranosyl-(1→2)-α-L-arabinopyranoside (alliospiroside C)	*D. marginata*	[49]
**41**	(22*R*,24*S*,25*S*)-Spirost-5-ene-1β,3β,24-triol 1-*O*-α-L-rhamnopyranosyl-(1→2)-α-L-arabinopyranoside	*D. marginata*	[49]
**42**	(22*R*,25*R*)-Spirost-5-ene-3β,7α-diol 3-*O*-α-L-rhamnopyranosyl-(1→2)-β-D-glucopyranoside (sansevierin A)	*S. ehrenbergii*	[57]
**43**	(14*R*,22*R*,25*R* and 14*R*,22*R*,25*S*)-Spirost-5-ene-3β,14-diol 3-*O*-α-L-rhamnoyranosyl-(1→2)-β-D-glucopyranoside (dracaenoside E)	*D. cochinchinensis*	[61]
**44**	(14*R*,22*R*,25*R* and 14*R*,22*R*,25*S*)-Spirost-5-ene-3β,14-diol 3-*O*-α-L-rhamnoyranosyl-(1→4)-β-D-glucopyranoside (dracaenoside E)	*D. cochinchinensis*	[61]
**45**	(17*S*,22*R*,25*R*)-Spirost-5-ene-3β,17-diol (pennogenin) 3-*O*-α-L-rhamnopyranosyl-(1→2)-β-D-glucopyranoside	*D. draco* *D. surculosa*	[29] [31]
**46**	(17*S*,22*R*,25*R*)-Spirost-5-ene-3β,17-diol (pennogenin) 3-*O*-α-L-rhamnopyranosyl-(1→3)-β-D-glucopyranoside (mannioside A)	*D. arborea* *D. mannii* *D. thalioides*	[27] [51] [32]
**47**	(17*S*,22*R*,25*R*)-Spirost-5-ene-3β,17-diol (pennogenin) 3-*O*-α-L-rhamnopyranosyl-(1→4)-β-D-glucopyranoside	*D. draco*	[29]

^a^ Both terresides A (**75**) and B (**23**) are known compounds; in reference [48] authors did not indicate whether one or both saponins were isolated from *D. angustifolia*.

**Table 4:** Spirostanol triglycosides isolated from *Dracaena* and *Sansevieria* species.

Number	Compound Name	Plant	References
**48**	(22*R*,24*S*,25*R*)-1-*O*-β-D-Fucopyranosyl-spirost-5-ene-1β,3β,24-triol 3-*O*-β-D-apiofuranosyl-(1→4)-β-D-glucopyranoside (surculoside A)	*D. sarculosa*	[31]
**49**	(22*S*,23*S*,24*S*)-24-*O*-β-D-Arabinopyranosyl-spirosta-5,25(27)-diene-1β,3β,23,24-tetrol 1-*O*-[(2,3,4-tri-*O*-acetyl)-α-L-rhamnopyranosyl-(1→2)]-α-L-arabinopyranoside (icodeside)	*D. draco*	[29]
**50**	(22*S*,23*S*,24*S*)-24-*O*-β-D-Fucopyranosyl-spirosta-5,25(27)-diene-1β,3β,23,24-tetraol 1-*O*-α-L-rhamnopyranosyl-(1→2)-α-L-arabinopyranoside (namonin C)	*D. angustifolia*	[26]
		*D. cambodiana*	[26,62]
**51**	(22*S*,23*S*,24*S*)-24-*O*-β-D-Fucopyranosyl-spirosta-5,25(27)-diene-1β,3β,23,24-tetraol 1-*O*-[(4-*O*-acetyl)-α-L-rhamnopyranosyl-(1→2)]-α-L-arabinopyranoside (namonin D)	*D. angustifolia*	[26]
		*D. cambodiana*	[56]
**52**	(22*S*,23*S*,24*S*)-24-*O*-β-D-Fucopyranosyl-spirosta-5,25(27)-diene-1β,3β,23,24-tetraol 1-*O*-[3,4-*O*-diacetyl)-α-L-rhamnopyranosyl-(1→2)]-α-L-arabinopyranoside (cambodracanoside A)	*D. cambodiana*	[56]
**53**	(22*S*,23*S*,24*S*)-24-*O*-β-D-Fucopyranosyl-spirosta-5,25(27)-diene-1β,3β,23,24-tetraol 1-*O*-[(2,3,4-*O*-triacetyl)-α-L-rhamnopyranosyl-(1→2)]-α-L-arabinopyranoside	*D. angustifolia* *D. draco*	[26] [28,53]
**54**	(22*S*,23*S*,24*S*,25*R*)-24-*O*-β-D-Fucopyranosyl-spirost-5-ene-1β,3β,23,24-tetraol 1-*O*-α-L-rhamnopyranosyl-(1→2)-α-L-arabinopyranoside (cambodracanoside B)	*D. cambodiana*	[56]
**55**	(22*R*,24*S*,25*R*)-24-*O*-β-D-Glucopyranosyl-spirost-5-en-1b,3β,24-triol 1-*O*-α-L-rhamnopyranosyl-(1→2)-β-D-fucopyranoside (surculoside C)	*D. sarculosa*	[31]
**56**	(22*R*)-Spirosta-5,25(27)-dien-3β-ol 3-*O*-α-L-rhamnoyranosyl-(1→2)-[β-D-glucopyranosyl-(1→3)]-β-D-glucopyranoside (dracaenoside I)	*D. cochinchinensis*	[61]
**57**	(22*R*)-Spirosta-5,25(27)-dien-3β-ol 3-*O*-α-L-rhamnopyranosyl-(1→2)-[α-L-rhamnopyranosyl-(1→4)]-β-D-glucopyranoside (sansevistatin 1)	*S. ehrenbergii*	[57]
**58**	(22*R*)-Spirosta-5,25(27)-diene-1β,3β-diol (neoruscogenin) 1-*O-*α-L-rhamnopyranosyl-(1→2)-[β-D-xylopyranosyl-(1→3)]-α-L-arabinopyranoside	*D. cambodiana*	[45]
		*D. fragrans* *(D. deisteliana)*	[27]
		*D. thalioides*	[32]
		*S. trifasciata* *(D. trifasciata)*	[33]
**59**	(22*R*)-Spirosta-5,25(27)-diene-1β,3β-diol (neoruscogenin) 1-*O*-α-L-rhamnopyranosyl-(1→2)-[β-D-xylopyranosyl-(1→3)]-β-D-glucopyranoside (trifasciatoside B)	*S. trifasciata* *(D. trifasciata)*	[33]
**60**	(22*S*,23*S*)-Spirosta-5,25(27)-diene-1β,3β,23-triol 1-*O*-α-L-rhamnopyranosyl-(1→2)-[β-D-xylopyranosyl-(1→3)]-α-L-arabinopyranoside	*D. draco*	[53]
		*S. trifasciata* *(D. trifasciata)*	[63]
**61**	(22*S*,23*S*)-Spirosta-5,25(27)-diene-1β,3β,23-triol 1-*O*-(2-*O*-acetyl)-α-L-rhamnopyranosyl-(1→2)-[β-D-xylopyranosyl-(1→3)]-α-L-arabinopyranoside (trifasciatoside K)	*S. trifasciata* *(D. trifasciata)*	[64]
**62**	(22*S*,23*S*)-Spirosta-5,25(27)-diene-1β,3β,23-triol 1-*O*-(3-*O*-acetyl)-α-L-rhamnopyranosyl-(1→2)-[β-D-xylopyranosyl-(1→3)]-α-L-arabinopyranoside (trifasciatoside L)	*S. trifasciata* *(D. trifasciata)*	[64]
**63**	(22*S*,23*S*)-Spirosta-5,25(27)-diene-1β,3β,23-triol 1-*O*-(4-*O*-acetyl)-α-L-rhamnopyranosyl-(1→2)-[β-D-xylopyranosyl-(1→3)]-α-L-arabinopyranoside	*S. trifasciata* *(D. trifasciata)*	[33,63]
**64**	(22*S*,23*S*)-Spirosta-5,25(27)-diene-1β,3β,23-triol 1-*O*-(2,3-di-*O*-acetyl)- α-L-rhamnopyranosyl-(1→2)-[β-D-xylopyranosyl-(1→3)]-α-L-arabinopyranoside	*S. trifasciata* *(D. trifasciata)*	[63]
**65**	(22*S*,23*S*,24*S*)-Spirosta-5,25(27)-diene-1β,3β,23,24-tetraol 1-*O*-α-L-rhamnopyranosyl-(1→2)-[β-D-xylopyranosyl-(1→3)]-α-L-arabinopyranoside	*D. cambodiana*	[62]
		*S. trifasciata* *(D. trifasciata)*	[33,63]
**66**	(22*S*,23*S*,24*S*)-Spirosta-5,25(27)-diene-1β,3β,23,24-tetraol 1-*O*-(2-*O*-acetyl)-α-L-rhamnopyranosyl-(1→2)-[β-D-xylopyranosyl-(1→3)]-α-L-arabinopyranoside (trifasciatoside M)	*S. trifasciata* *(D. trifasciata)*	[64]
**67**	(22*S*,23*S*,24*S*)-Spirosta-5,25(27)-diene-1β,3β,23,24-tetraol 1-*O*-(3-*O*-acetyl)-α-L-rhamnopyranosyl-(1→2)-[β-D-xylopyranosyl-(1→3)]-α-L-arabinopyranoside (trifasciatoside N)	*S. trifasciata* *(D. trifasciata)*	[64]
**68**	(22*S*,23*S*,24*S*)-Spirosta-5,25(27)-diene-1β,3β,23,24-tetraol 1-*O*-(4-*O*-acetyl)-α-L-rhamnopyranosyl-(1→2)-[β-D-xylopyranosyl-(1→3)]-α-L-arabinopyranoside	*S. trifasciata* *(D. trifasciata)*	[33,63]
**69**	(22*S*,23*S*,24*S*)-Spirosta-5,25(27)-diene-1β,3β,23,24-tetraol 1-*O*-(2,3-di-*O*-acetyl)-α-L-rhamnopyranosyl-(1→2)-[β-D-xylopyranosyl-(1→3)]-α-L-arabinopyranoside	*S. trifasciata* *(D. trifasciata)*	[33,63]
**70**	(22*S*,23*S*,24*S*)-Spirosta-5,25(27)-diene-1β,3β,23,24-tetraol 1-*O*-(2,4-di-*O*-acetyl)-α-L-rhamnopyranosyl-(1→2)-[β-D-xylopyranosyl-(1→3)]-α-L-arabinopyranoside (trifasciatoside G)	*S. trifasciata* *(D. trifasciata)*	[33]
**71**	(22*S*,23*S*,24*S*)-Spirosta-5,25(27)-diene-1β,3β,23,24-tetraol 1-*O*-(3,4-di-*O*-acetyl)-α-L-rhamnopyranosyl-(1→2)-[β-D-xylopyranosyl-(1→3)]-α-L-arabinopyranoside (trifasciatoside H)	*S. trifasciata* *(D. trifasciata)*	[33]
**72**	(22*S*,23*S*,24*S*)-Spirosta-5,25(27)-diene-1β,3β,23,24-tetraol 1-*O*-(2,3,4-tri-*O*-acetyl)-α-L-rhamnopyranosyl-(1→2)-[β-D-xylopyranosyl-(1→3)]-α-L-arabinopyranoside	*D. angustifolia*	[26]
		*D. thalioides*	[32]
		*S. trifasciata* *(D. trifasciata)*	[63]
**73**	(22*R*,25*R*)-5β-Spirostan-3-β-ol (smilagenin) 3-*O*-β-D-galactopyranosyl-(1‴→4″)-β-D-galactopyranosyl-(1″→3′)-β-D-glucopyranoside	*D. ombet*	[30]
**74**	(22*R*,25*R*)-5β-spirostan-3-β-ol (smilagenin) 3-*O*-β-D-galactopyranosyl-(1‴→4″)-β-D-glucopyranosyl-(1′’→3′)-β-D-glucopyranoside	*D. ombet*	[30]
**75**	(22*R*,25*R*)-5α-Spirostan-3-β-ol-12-one 3-*O*-β-D-galactopyranosyl-(1‴→4″)-β-D-glucopyranosyl-(1″→2′)-β-D-glucopyranoside (terreside A) ^a^	*D. angustifolia*	[48]
**76**	(22*R*,25*R*)-Spirosta-3β,5α,6β-triol 3-*O*-b-L-rhamnopyranosyl-(1→2)-[α-L-rhamnopyranosyl-(1→3)]-β-D-glucopyranoside (cambodianoside G)	*D. cambodiana*	[62]
**77**	(22*R*,25*S*)-Spirost-5-ene-1β,3β-diol [(*S*)-ruscogenin] 1-*O*-α-L-rhamnopyranosyl-(1→2)-[β-D-xylopyranosyl-(1→3)]-α-L-arabinopyranoside	*D. angustifolia*	[48]
		*D. marginata*	[49]
		*D. thalioides*	[32]
		*S. cylindrica*	[47]
		*S. trifasciata* *(D. trifasciata)*	[33]
**78**	(22*R*,25*R*)-Spirost-5-ene-1β,3β-diol [(*R*)-ruscogenin] 1-*O*-α-L-rhamnopyranosyl-(1→2)-[β-D-xylopyranosyl-(1→3)]-β-D-glucopyranoside (trifasciatoside C)	*S. trifasciata* *(D. trifasciata)*	[33]
**79**	(22*R*,25*S*)-Spirost-5-ene-1b,3β-diol [(*S*)-ruscogenin] 1-*O*-α-L-rhamnopyranosyl-(1→2)-[β-D-xylopyranosyl-(1→3)]-β-D-glucopyranoside (trifasciatoside D)	*D. cambodiana*	[45]
		*S. trifasciata* *(D. trifasciata)*	[33]
**80**	(22*R*,25*R*)-Spirost-5-ene-1β,3β-diol [(*R*)-ruscogenin] 1-*O*-α-L-rhamnopyranosyl-(1→2)-[β-D-xylopyranosyl-(1→3)]-β-D-xylopyranoside	*D. thalioides*	[32]
**81**	(22*R*,24*S*,25*R*)-Spirost-5-ene-1β,3β,24-triol 1-*O*-α-L-rhamnopyranosyl-(1→2)-[β-D-xylopyranosyl-(1→3)]-α-L-arabinopyranoside	*D. marginata*	[49]
**82**	(22*S*,23*S*,24*S,*25*S*)-Spirost-ene-1β,3β,23,24-tetraol 1-*O*-α-L-rhamnopyranosyl-(1→2)*-*[β-D-xylopyranosyl-(1→3)]-α-L-arabinopyranoside	*D. concinna*	[59]
**83**	(22*R*,25*S*)-Spirost-5-ene-1β,3β-diol [(*S*)-ruscogenin] 3-*O*-α-L-rhamnopyranosyl-(1→2)-[α-L-rhamnopyranosyl-(1→3)]-β-D-glucopyranoside (drangustoside A)	*D. angustifolia*	[58]
**84**	(22*R*,25*R*)-Spirost-5-en-3β-ol (diosgenin) 3-*O*-α-L-rhamnopyranosyl-(1→2)-[α-L-arabinopyranosyl-(1→4)]-β-D-glucopyranoside (sansevistatin 2)	*S. ehrenbergii*	[57]
**85**	(22*R*,25*R*)-Spirost-5-en-3β-ol (diosgenin) 3-*O*-α-L-rhamnopyranosyl-(1→2)-[β-D-glucopyranosyl-(1→3)]-β-D-glucopyranoside (gracillin)	*D. concinna* *D. draco* *D. viridiflora*	[,^59^] [28] [50]
**86**	(22*R*,25*R* and 22*R*,25*S*)-Spirost-5-en-3β-ol 3-*O*-α-L-rhamnopyranosyl-(1→2)-[β-D-glucopyranosyl-(1→3)]-β-D-glucopyranoside	*D. cochinchinensis*	[61]
**87**	(22*R*,25*R*)-Spirost-5-en-3β-ol (diosgenin) 3-*O*-α-L-rhamnopyranosyl-(1→2)-[α-L-rhamnopyranosyl-(1→3)]-β-D-glucopyranoside	*D. cambodiana*	[45]
**88**	(22*R*,25*R*)-Spirost-5-en-3β-ol (diosgenin) 3-*O*-α-L-rhamnopyranosyl-(1→2)-[α-L-rhamnopyranosyl-(1→4)]-β-D-glucopyranoside (dioscin)	*D. concinna*	[59]
		*D. draco*	[28,29,55]
		*D. viridiflora*	[50]
		*S. ehrenbergii*	[57]
**89**	(22*R*,25*R* and 22*R*,25*S*)-Spirost-5-en-3β-ol 3-*O*-α-L-rhamnopyranosyl-(1→2)-[α-L-rhamnopyranosyl-(1→4)]-β-D-glucopyranoside	*D. cochinchinensis*	[61]
**90**	(22*S*,25*S*)-Spirost-5-en-3β-ol 3-*O*-α-L-rhamnopyranosyl-(1→2)-[α-L-rhamnopyranosyl-(1→4)]-β-D-glucopyranoside (borassoside E)	*D. marginata*	[49]
**91**	(22*R*,25*R*)-Spirost-5-en-3β-ol (diosgenin) 3-*O*-α-L-rhamnopyranosyl-(1→2)-[β-D-xylopyranosyl-(1→4)]-β-D-glucopyranoside	*S. ehrenbergii*	[57]
**92**	(14*R*,22*R*,25*R* and 14*R*,25*S*)-Spirost-5-ene-3β,14-diol 3-*O*-α-L-rhamnoyranosyl-(1→2)-[β-D-glucopyranosyl-(1→)]-β-D-glucopyranoside (dracaenoside H)	*D. cochinchinensis*	[61]
**93**	(14*R*,22*R*,25*R* and 14*R*,22*R*,25*S*)-Spirost-5-ene-3β,14-diol 3-*O*-α-L-rhamnoyranosyl-(1→2)-[α-L-rhamnopyranosyl-(1→4)]-β-D-glucopyranoside (dracaenoside G)	*D. cochinchinensis*	[61]
**94**	(14*R*,22*R*,24*S*,25*R*)-Spirost-5-en-3β,14,24-triol 3-*O*-α-L-rhamnopyranosyl-(1→2)-[β-D-glucopyranosyl-(1→3)]-β-D-glucopyranoside (dracaenoside L)	*D. cochinchinensis*	[61]
**95**	(14*R*,22*R*,24*S*,25*R*)-Spirost-5-en-3β,14,24-triol 3-*O*-α-L-rhamnopyranosyl-(1→2)-[α-L-rhamnopyranosyl-(1→4)]-β-D-glucopyranoside (dracaenoside K)	*D. cochinchinensis*	[61]
**96**	(14*R*,22*R*,25*S*)-Spirost-5-ene-3β,14,27-triol 3-*O*-α-L-rhamnopyranosyl--(1→2)*-*[β-D-glucopyranosyl-(1→3)]-β-D-glucopyranoside (dracaenoside J)	*D. cochinchinensis*	[61]
**97**	(17*S*,22*R*,25*R*)-Spirost-5-ene-3β,17-diol (pennogenin) 3-*O*-α-L-rhamnopyranosyl-(1→2)-[α-L-rhamnopyranosyl-(1→3)]-β-D-glucopyranoside (spiroconazole A)	*D. arborea*	[27]
		*D. cambodiana*	[45]
		*D. mannii*	[51,65,66]
		*D. thalioides*	[32]
**98**	(17*S*,22*R*,25*R*)-Spirost-5-ene-3β,17-diol (pennogenin) 3-*O*-α-L-rhamnopyranosyl-(1→2)-[α-L-rhamnopyranosyl-(1→3)]-(4-*O*-acetyl)-β-D-glucopyranoside	*D. thalioides*	[32]
**99**	(17*S*,22*R*,25*R*)-Spirost-5-ene-3β,17-diol (pennogenin) 3-*O*-α-L-rhamnopyranosyl-(1→2)-[α-L-rhamnopyranosyl-(1→3)]-(6-*O*-acetyl)-β-D-glucopyranoside (arboreasaponin A)	*D. arborea*	[27]
**100**	(17*S*,22*R*,25*R*)-Spirost-5-ene-3β,17-diol (pennogenin) 3-*O*-α-L-rhamnopyranosyl-(1→2)-[α-L-rhamnopyranosyl-(1→3)]-(4,6-*O*-diacetyl)-β-D-glucopyranoside	*D. thalioides*	[32]
**101**	(17*S*,22R,25*R*)-Spirost-5-en-3β,17-diol (pennogenin) 3-*O*-α-L-rhamnopyranosyl-(1→2)-[α-L-rhamnopyranosyl-(1→4)]-β-D-glucopyranoside (pennogenin 3-*O*-β-chacotrioside)	*D. draco* *D. surculosa*	[29] [31]
**102**	(17*S*,22*R*,24*R*,25*S*)-Spirost-5-ene-3β,17,24-triol 3-*O*-α-L-rhamnopyranosyl-(1→2)-[α-L-rhamnopyranosyl-(1→3)]-β-D-glucopyranoside (arboreasaponin B)	*D. arborea*	[27]
**103**	(22*R*,24*S*,25*S*)-Spirost-5-ene-3β,24,27-triol 3-*O*-α-L-rhamnopyranosyl-(1→2)-[α-L-rhamnopyranosyl-(1→3)]-β-D-glucopyranoside (cambodianoside B)	*D. cambodiana*	[45]
**104**	(22*R*,25*S*)-Spirost-5-ene-3β,27-diol 3-*O*-α-L-rhamnopyranosyl-(1→2)-[α-L-rhamnopyranosyl-(1→3)]-β-D-glucopyranoside (cambodianoside C)	*D. cambodiana*	[45]

^a^ Both terresides A (**75**) and B (**23**) are known compounds; in reference [48] authors did not indicate whether one or both saponins were isolated from *D. angustifolia*.

**Table 5:** Spirostanol tetraglycosides isolated from *Dracaena* and *Sansevieria* species.

Number	Compound Name	Source	References
**105**	(22*S*,23*S*,24*S*)-24-*O*-α-L-Arabinopyranosyl-spirosta-5,25(27)-diene-1β,3β,23,24-tetraol 1-*O*-β-D-xylopyranosyl-(1→2)-[α-L-rhamnopyranosyl-(1→3)]-β-D-fucopyranoside (deistelianoside B)	*D. fragrans* *(D. deisteliana)*	[27]
**106**	(22*S*,23*S*,24*S*)-24-*O*-β-D-Fucopyranosyl-spirosta-5,25(27)-diene-1β,3β,23,24-tetraol 1-*O*-α-L-rhamnopyranosyl-(1→2)-[β-D-xylopyranosyl-(1→3)]-*O*-α-L-arabinopyranoside	*D. cambodiana*	[62]
**107**	(22*S*,23*S*,24*S*)-24-*O*-β-D-Fucopyranosyl-spirosta-5,25(27)-diene-1β,3β,23,24-tetraol 1-*O*-(4-*O*-acetyl)-α-L-rhamnopyranosyl-(1→2)-[β-D-xylopyranosyl-(1→3)]-α-L-arabinopyranoside	*D. cambodiana*	[62]
		*S. trifasciata* *(D. trifasciata)*	[33,63]
**108**	(22*S*,23*S*,24*S*)-24-*O*-β-D-Fucopyranosyl-spirosta-5,25(27)-diene-1β,3β,23,24-tetraol 1-*O*-(2,3-di-*O*-acetyl)-α-L-rhamnopyranosyl-(1→2)-[β-D-xylopyranosyl-(1→3)]-α-L-arabinopyranoside	*D. thalioides*	[32]
		*S. trifasciata* *(D. trifasciata)*	[33,63]
**109**	(22*S*,23*S*,24*S*)-24-*O*-β-D-Fucopyranosyl-spirosta-5,25(27)-diene-1β,3β,23,24-tetraol 1-*O*-(2,4-di-O-acetyl)-α-L-rhamnopyranosyl-(1→2)-[β-D-xylopyranosyl-(1→3)]-α-L-arabinopyranoside (trifasciatoside I)	*S. trifasciata* *(D. trifasciata)*	[33]
**110**	(22*S*,23*S*,24*S*)-24-*O*-β-D-Fucopyranosyl-spirosta-5,25(27)-diene-1β,3β,23,24-tetraol 1-*O*-(3,4-di-O-acetyl)-α-L-rhamnopyranosyl-(1→2)-[β-D-xylopyranosyl-(1→3)]-α-L-arabinopyranoside (trifasciatoside J)	*S. trifasciata* *(D. trifasciata)*	[33]
**111**	(22*S*,23*S*,24*S*)-24-*O*-β-D-Fucopyranosyl-spirosta-5,25(27)-diene-1β,3β,23,24-tetraol 1-*O*-(2,3,4-tri-*O*-acetyl)-α-L-rhamnopyranosyl-(1→2)-[β-D-xylopyranosyl-(1→3)]-α-L-arabinopyranoside	*D. angustifolia*	[26]
		*D. thalioides*	[32]
		*S. trifasciata* *(D. trifasciata)*	[33,63]
**112**	(22*S*,23*S*,24*S*)-24-*O*-β-D-Fucopyranosyl-spirosta-5,25(27)-diene-1β,3β,23,24-tetraol 1-*O*-(2,3,4-tri-*O*-acetyl)-α-L-rhamnopyranosyl-(1→2)-[(3-*O*-acetyl)-β-D-xylopyranosyl-(1→3)]-α-L-arabinopyranoside (namonin A)	*D. angustifolia*	[26]
		*D. thalioides*	[32]
**113**	(22*S*,23*S*,24*S*)-24-*O*-β-D-Fucopyranosyl-spirosta-5,25(27)-diene-1β,3β,23,24-tetraol 1-*O*-(2,3,4-tri-*O*-acetyl)-α-L-rhamnopyranosyl-(1→2)-[(4-*O*-acetyl)-β-D-xylopyranosyl-(1→3)]-α-L-arabinopyranoside (namonin B)	*D. angustifolia*	[26]
**114**	(22*S*,23*S*,24*S*)-24-*O*-β-D-Glucopyranosyl-spirosta-5,25(27)-diene-1β,3β,23,24-tetraol 1-*O*-(2,3,4-tri-*O*-acetyl)-α-L-rhamnopyranosyl-(1→2)-[β-D-xylopyranosyl-(1→3)]-α-L-arabinopyranoside	*D. thalioides*	[32]
		*S. trifasciata* *(D. trifasciata)*	[63]
**115**	(22*S*,23*S*,24*S*)-24-*O*-β-D-Glucopyranosyl-spirosta-5,25(27)-diene-1β,3β,23,24-tetraol 1-*O*-(2,3,4-tri-*O*-acetyl)-α-L-rhamnopyranosyl-(1→2)-[(3-*O*-acetyl)-β-D-xylopyranosyl-(1→3)]-α-L-arabinopyranoside	*D. thalioides*	[32]
**116**	(22*S*,23*S*,24*S*)-24-*O*-α-L-Rhamnopyranosyl-spirosta-5,25(27)-diene-1β,3β,23,24-tetraol 1-*O*-(2,3,4-tri-*O*-acetyl)-α-L-rhamnopyranosyl-(1→2)-[β-D-xylopyranosyl-(1→3)]-*O*-α-L-arabinopyranoside	*S. trifasciata* *(D. trifasciata)*	[63]
**117**	(22*S*,23*S*,24*S*,25*S*)-24-*O*-β-D-Fucopyranosyl-spirost-5-ene-1β,3β,23,24-tetraol 1-*O*-α-L-rhamnopyranosyl-(1→2)-[β-D-xylopyranosyl-(1→3)]-*O*-α-L-arabinopyranoside	*D. concinna*	[59]
**118**	(22*S*,23*S*,24*S*,25*S*)-24-*O*-β-D-Fucopyranosyl-spirost-5-ene-1β,3β,23,24-tetraol 1-*O*-(2,3,4-tri-*O*-acetyl)-α-L-rhamnopyranosyl-(1→2)-[β-D-xylopyranosyl-(1→3)]-*O*-α-L-arabinopyranoside	*D. thalioides*	[32]

**Table 6:** Furostanol diglycosides isolated from *Dracaena* and *Sansevieria* species.

Number	Compound Name	Source	References
**119**	(22*S*,25*S*)-Furost-5-en-22(25)-epoxy-1β,3β,26-triol 26-*O*-β-D-glucopyranosyl-(1→2)-β-D-glucopyranoside (cambodianoside E)	*D. cambodiana*	[45]
**120**	(22*R*,25*S*)-26-*O*-β-D-Glucopyranosyl-22-methoxy-3α,5α-cyclofurostane-1β,6β,26-triol 1-*O*-β-D-fucopyranoside	*D. surculosa*	[52]
**121**	(22*R*,25*S*)-26-*O*-β-D-Glucopyranosyl-22-methoxy-3α,5α-cyclofurostane-1β,6β,26-triol 1-*O*-β-D-glucopyranoside	*D. surculosa*	[52]
**122**	(22*R*,25*S*)-26-*O*-β-D-Glucopyranosyl-22-methoxy-furost-5-ene-1β,3β,26-triol 1-*O*-β-D-fucopyranoside	*D. surculosa*	[31]
**123**	(22*R*,25*S*)-26-*O*-β-D-Glucopyranosyl-22-methoxy-furost-5-ene-1β,3β,26-triol 1-*O*-β-D-glucopyranoside	*D. surculosa*	[31]

**Table 7:** Furostanol triglycosides isolated from *Dracaena* and *Sansevieria* species.

Number	Compound Name	Source	References
**124**	(22*R*,25*R*)-Furost-5-ene-3β,22,26-triol 3-*O*-α-L-rhamnopyranosyl-(1→2)-[β-D-glucopyranosyl-(1→3)]-β-D-glucopyranoside (icogenin)	*D. draco*	[55]
**125**	(14*R*,22*S*,25*S*)-Furost-5-en-22(25)-epoxy-3β,14,26,27-tetraol 3-*O*-α-L-rhamnopyranosyl-(1→2)-[α-L-rhamnopyranosyl-(1→4)]-β-D-glucopyranoside (dracaenoside R)	*D. cochinchinensis*	[61]
**126**	(22*R*)-26-*O*-β-D-Glucopyranosyl-furosta-5,25(27)-diene-1β,3β,22,26-tetraol 1-*O*-α-L-rhamnopyranosyl-(1→2)-α-L-arabinopyranoside	*S. trifasciata* *(D. trifasciata)*	[33]
**127**	(22*R*)-26-*O*-β-D-Glucopyranosyl-furosta-5,20(22),25(27)-triene-1β,3β,22,26-tetraol 1-*O*-α-L-rhamnopyranosyl-(1→2)-α-L-arabinopyranoside	*D. angustifolia* *D. cambodiana*	[26] [56]
**128**	(22*R*,25*S*)-26-*O*-β-D-Glucopyranosyl-furost-5-ene-1β,3β,22,26-tetraol 1-*O*-α-L-rhamnopyranosyl-(1→2)-α-L-arabinopyranoside (alliofuroside A)	*D. marginata*	[49]
**129**	(22*R*,25*S*)-26-*O*-β-D-Glucopyranosyl-furost-5-ene-1β,3β,22,26-tetraol 1-*O*-α-L-rhamnopyranosyl-(1→2)-(4-*O-*sulfo)-α-L-arabinopyranoside	*D. marginata*	[49]
**130**	(22*R*,25*S*)-26-*O*-β-D-Glucopyranosyl-3-*O*-sulfo-furost-5-ene-1β,3β,22,26-tetraol 1-*O*-α-L-rhamnopyranosyl-(1→2)-(3,4-*O*-diacetyl)-α-L-arabinopyranoside sodium salt	*D. thalioides*	[67]
**131**	(14*R*,22ξ,25*R* + 14*R*,22ξ,25*S*)-26-*O*-β-D-Glucopyranosyl-furost-5-ene-3β,14,22,26-tetraol 3-*O*-α-L-rhamnopyranosyl-(1→2)-β-D-glucopyranoside (ophipojaponin A + dracaenoside N)	*D. cochinchinensis*	[61]
**132**	(14*R*, 22ξ,25*R* and 14*R*,22ξ,25*S*)-26-*O*-β-D-Glucopyranosyl-furost-5-ene-3β,14,22,26-tetraol 3-*O*-α-L-rhamnopyranosyl-(1→4)-β-D-glucopyranoside (dracaenoside M)	*D. cochinchinensis*	[61]
**133**	(22ξ)-26-*O*-β-D-Glucopyranosyl-22-methoxy-furosta-5,25(27)-diene-1β,3β,26-triol 1-*O*-α-L-rhamnopyranosyl-(1→2)-α-L-arabinopyranoside	*D. cambodiana* *D. draco*	[56] [53,]
**134**	(22*R*)*-*26-*O*-β-D-Glucopyranosyl-22-methoxy-furosta-5,25(27)-diene-1β,3β,26-triol 1-*O*-α-L-rhamnopyranosyl-(1→2)-α-L-arabinopyranoside	*S. trifasciata* *(D. trifasciata)*	[63]
**135**	(22*R*,25*S*)-26-*O*-β-D-Glucopyranosyl-22-methoxy-furost-5-ene-1β,3β,26-triol 1-*O*-α-L-rhamnopyranosyl-(1→2)-β-D-fucopyranoside	*D. surculosa*	[31]
**136**	(22ξ)-26-*O*-β-D-Glucopyranosyl-22-methoxy-5α-furost-25(27)-ene-1β,3α,26-triol 1-*O*-α-L-rhamnopyranosyl-(1→2)-β-D-fucopyranoside	*D. concinna*	[59]
**137**	(22ξ)-26-*O*-β-D-Glucopyranosyl-22-methoxy-5α-furost-25(27)-ene-1β,3α,4α,26-tetraol 1-*O*-α-L-rhamnopyranosyl-(1→2)-β-D-fucopyranoside	*D. concinna*	[59]
**138**	(22ξ)-26-*O*-β-D-Glucopyranosyl-22-methoxy-5α-furost-25(27)-ene-1β,3β,4α,26-tetraol 1-*O*-α-L-rhamnopyranosyl-(1→2)-β-D-fucopyranoside	*D. concinna*	[59]
**139**	(22*R*,25*R*)-26-*O*-α-L-Rhamnopyranosyl-furost-5-ene-3β,22,26-triol 3-*O*-α-L-rhamnopyranosyl-(1→4)-β-D-glucopyranoside (afromontoside)	*D. afromontana*	[68]

**Table 8:** Furostanol tetraglycosides isolated from *Dracaena* and *Sansevieria* species.

Number	Compound Name	Source	References
**140**	(25*R*)-26-*O*-β-D-Glucopyranosyl-furosta-5,20(22)-diene-1β,3β,26-triol 1-*O*-α-L-rhamnopyranosyl-(1→2)-[β-D-xylopyranosyl-(1→3)]-(4-O-acetyl)-α-L-arabinopyranoside (namonin E)	*D. angustifolia*	[26]
**141**	(14*R*,25*R* and 14*R*,25*S*)-26-*O*-β-D-Glucopyranosyl-furosta-5,20(22)-diene-3β,14,26-triol 3-*O*-α-L-rhamnopyranosyl-(1→2)-[β-D-glucopyranosyl-(1→3)]-β-D-glucopyranoside (dracaenoside Q)	*D. cochinchinensis*	[61]
**142**	(22*R*)-26-*O*-β-D-Glucopyranosyl-furosta-5,25(27)-diene-1β,3β,22,26-tetraol 1-*O*-(4-*O*-acetyl)-α-L-rhamnopyranosyl-(1→2)-[β-D-xylopyranosyl-(1→3)]-α-L-arabinopyranoside	*S. trifasciata* *(D. trifasciata)*	[33]
**143**	(22ξ)-26-*O*-β-D-Glucopyranosyl-22-methoxy-furosta-5,25(27)-diene-1β,3β,26-triol 1-*O*-α-L-rhamnopyranosyl-(1→2)-[β-D-xylopyranosyl-(1→3)]-α-L-arabinopyranoside	*D. angustifolia*	[48]
**144**	(22ξ)-26-*O*-β-D-Glucopyranosyl-22-methoxy-furosta-5,25(27)-diene-1β,3β,26-triol 1-*O*-α-L-rhamnopyranosyl-(1→2)-[β-D-xylopyranosyl-(1→3)]-β-D-fucopyranoside	*D. concinna*	[59]
**145**	(22*S*,25*S*)-26-*O*-β-D-Glucopyranosyl-furost-5-en-22(25)-epoxy-1β,3β,26-triol 1-*O*-α-L-rhamnopyranosyl-(1→2)-[β-D-xylopyranosyl-(1→3)]-β-D-glucopyranoside (trifasciatoside A)	*S. trifasciata* *(D. trifasciata)*	[33]
**146**	(22*R*,25*R*)-26-*O*-β-D-Glucopyranosyl-furost-5-ene-1β,3β,22,26-tetraol 1-*O*-α-L-rhamnopyranosyl-(1→2)-[β-D-xylopyranosyl-(1→3)]-α-L-arabinopyranoside	*S. cylindrica*	[47]
**147**	(22*R*,25*S*)-26-*O*-β-D-Glucopyranosyl-furost-5-ene-1β,3β,22,26-tetraol 1-*O*-α-L-rhamnopyranosyl-(1→2)-[β-D-xylopyranosyl-(1→3)]-α-L-arabinopyranoside	*D. thalioides*	[67]
**147a** ^a^	26-*O*-β-D-Glucopyranosyl-furost-5-ene-1β,3β,22,26-tetraol 1-*O*-α-L-rhamnopyranosyl-(1→2)-[β-D-xylopyranosyl-(1→3)]-α-L-arabinopyranoside	*D. angustifolia*	[48]
**148**	(22*R*,25*S*)-26-*O*-β-D-Glucopyranosyl-furost-5-ene-1β,3β,22,26-tetraol 1-*O*-α-L-rhamnopyranosyl-(1→2)-[β-D-xylopyranosyl-(1→3)]-β-D-glucopyranoside (trifasciatoside E)	*S. trifasciata* *(D. trifasciata)*	[33]
**149**	(22*R*,25*R*)-26-*O*-β-D-Glucopyranosyl-furost-5-ene-1β,3β,22,26-tetraol 1-*O*-α-L-rhamnopyranosyl-(1→2)-[β-D-xylopyranosyl-(1→3)]-β-D-xylopyranoside	*D. thalioides*	[67]
**150**	(22*R*,25*S*)-26-*O*-β-D-Glucopyranosyl-furost-5-ene-1β,3β,22,26-tetraol 1-*O*-α-L-rhamnopyranosyl-(1→2)-[α-L-rhamnopyranosyl-(1→4)]-β-D-glucopyranoside	*D. marginata*	[49]
**151**	(22*R*,25*S*)-26-*O*-β-D-Glucopyranosyl-22-methoxy-furost-5-ene-1β,3β,26-triol 1-*O*-α-L-rhamnopyranosyl-(1→2)-[β-D-xylopyranosyl-(1→3)]-β-D-glucopyranoside (trifasciatoside F)	*S. trifasciata* *(D. trifasciata)*	[33]
**152**	(12*R*,15*R*,22*R*,25*S*)-26-*O*-β-D-Glucopyranosyl-12,15-di-*O*-α-L-rhamnopyranosyl-furost-5-ene-3β,12,15,22,26-pentaol 3-*O-*β-D-glucopyranoside	*S. cylindrica*	[69]
**153**	(14*R*,22ξ,25*R* and 14*R*,22ξ,25*S*)-26-*O*-β-D-Glucopyranosyl-furost-5-ene-3β,14,22,26-tetraol 3-*O*-α-L-rhamnopyranosyl-(1→2)-[β-D-glucopyranosyl-(1→3)]-β-D-glucopyranoside (dracaenoside P)	*D. cochinchinensis*	[61]
**154**	(14*R*,22ξ,25*R* and 14*R*,22ξ,25*S*)-26-*O*-β-D-Glucopyranosyl-furost-5-ene-3β,14,22,26-tetraol 3-*O*-α-L-rhamnopyranosyl-(1→2)-[α-L-rhamnopyranosyl-(1→4)]-β-D-glucopyranoside (dracaenoside O)	*D. cochinchinensis*	[61]
**155**	(22*R*,25*R*)-26-*O*-β-D-Glucopyranosyl-furost-5-ene-3β,22,26-triol 3-*O*-α-L-rhamnopyranosyl-(1→2)-[β-D-glucopyranosyl-(1→3)]-β-D-glucopyranoside	*D. draco*	[28]
**156**	(22ξ,25*R* and 22ξ,25*S*)-26-*O*-β-D-Glucopyranosyl-furost-5-ene-3β,22,26-triol 3-*O*-α-L-rhamnopyranosyl-(1→2)-[β-D-glucopyranosyl-(1→3)]-β-D-glucopyranoside	*D. cochinchinensis*	[61]
**157**	(22*R*,25*R*)-26-*O*-β-D-Glucopyranosyl-furost-5-ene-3β,22,26-triol 3-*O*-α-L-rhamnopyranosyl-(1→2)-[α-L-rhamnopyranosyl-(1→4)]-β-D-glucopyranoside	*D. draco*	[28]
**158**	(22*R*,25*S*)-26-*O*-β-D-Glucopyranosyl-furost-5-ene-3β,22,26-triol 3-*O*-α-L-rhamnopyranosyl-(1→2)-[α-L-rhamnopyranosyl-(1→4)]-β-D-glucopyranoside (protoneodioscin)	*D. marginata*	[49]
**159**	(22ξ,25*R* and 22ξ,25*S*)-26-*O*-β-D-Glucopyranosyl-furost-5-ene-3β,22,26-triol 3-*O*-α-L-rhamnopyranosyl-(1→2)-[α-L-rhamnopyranosyl-(1→4)]-β-D-glucopyranoside	*D. cochinchinensis*	[61]
**160**	(22ξ,25*R*)-26-*O*-β-D-Glucopyranosyl-22-methoxy-furost-5-ene-3β,26-diol 3-*O*-α-L-rhamnopyranosyl-(1→2)-[β-D-glucopyranosyl-(1→3)]-β-D-glucopyranoside	*D. concinna*	[59]
**161**	(22*R*,25*R*)-26-*O*-β-D-Glucopyranosyl-22-methoxy-furost-5-ene-3β,26-diol 3-*O*-α-L-rhamnopyranosyl-(1→2)-[α-L-rhamnopyranosyl-(1→4)]-β-D-glucopyranoside (methyl protodioscin)	*D. viridiflora*	[50]
**162**	(22*R*,25*S*)-26-*O*-β-D-Glucopyranosyl-22-methoxy-furost-5-ene-3β,26-diol 3-*O*-α-L-rhamnopyranosyl-(1→2)-[α-L-rhamnopyranosyl-(1→4)]-β-D-glucopyranoside (methyl protoneodioscin)	*D. marginata*	[49]
**163**	(22ξ,25*R*)-26-*O*-β-D-Glucopyranosyl-22-methoxy-furost-5-ene-3β,26-diol 3-*O*-α-L-rhamnopyranosyl-(1→2)-[α-L-rhamnopyranosyl-(1→4)]-β-D-glucopyranoside	*D. concinna*	[59]

^a^ Unassigned stereochemistry at C-22 and C-25.

**Table 9:** Miscellaneous steroidal saponins isolated from *Dracaena* and *Sansevieria* species.

Number	Compound Name	Source	References
**164**	Cambodianoside A	*D. cambodiana*	[45]
**165**	Cambodianoside D	*D. cambodiana*	[45]
**166**	Dracaenoside A	*D. cochinchinensis*	[70]
**167**	Dracaenoside B	*D. cochinchinensis*	[70]
**168**	Pregna-5,16-diene-1β,3β-diol-20-one 1-*O-*α-L-rhamnopyranosyl-(1→2)-α-L-arabinopyranoside	*D. angustifolia*	[26]
		*D. cambodiana*	[62]
		*S. trifasciata* *(D. trifasciata)*	[71]
**169**	Pregna-5,16-diene-1β,3β-diol-20-one 1-*O-*α-L-rhamnopyranosyl-(1→2)*-*[β-D-xylopyranosyl-(1→3)]-α-L-arabinopyranoside	*S. trifasciata* *(D. trifasciata)*	[33,71]
**170**	Pregna-5,16-diene-1β,3β-diol-20-one 1-*O-*α-L-rhamnopyranosyl-(1→2)-[β-D-xylopyranosyl-(1→3)]-β-D-glucopyranoside	*S. trifasciata* *(D. trifasciata)*	[33,71]
**171**	Pregna-5,16-diene-1β,3β-diol-20-one 1-*O-*α-L-rhamnopyranosyl-(1→2)-[β-D-xylopyranosyl-(1→3)]-6-*O*-acetyl-β–D-glucopyranoside	*S. trifasciata* *(D. trifasciata)*	[71]
**172**	Pregna-5,16-diene-3β,14α-diol-20-one 3-*O-*α-L-rhamnopyranosyl-(1→2)-[β-D-glucopyranosyl-(1→3)]-β-D-glucopyranoside (dracaenoside D)	*D. cochinchinensis*	[72]
**173**	Pregna-5,16-dien-3β-ol-20-one 3-*O-*α-L-rhamnopyranosyl-(1→2)-[α-L-rhamnopyranosyl-(1→3)]-β-D-glucopyranoside	*D. cambodiana*	[45]
**174**	Pregna-5,16-diene-3β,14α-diol-20-one 3-*O-*α-L-rhamnopyranosyl-(1→2)-[α-L-rhamnopyranosyl-(1→4)]-β-D-glucopyranoside (dracaenoside C)	*D. cochinchinensis*	[72]
**175**	(16*S*)-1-*O*-α-L-Rhamnopyranosyl-(1→2)-*O*-α-L-arabinopyranosyl-pregn-5-ene-1β,3β,16-triol 16-*O*-(4-β-D-glucopyranosyloxymethyl)-pent-4-enoate (namonin F)	*D. angustifolia*	[26]
**176**	(25*R*)-26-*O*-β-D-Glucopyranoyl-cholesta-5,17-dien-3β-ol-16,22-dione 3-*O*-α-L-rhamnopyranosyl-(1→2)-[α-L-rhamnopyranosyl-(1→3)]-β-D-glucopyranoside	*D. cambodiana*	[45]
**177**	1-*O*-α-L-Rhamnopyranosyl-5α-cholesta-1β,3β,16β-triol-7,23-dione 16-β-D-glucopyranoside (concinnasteoside A)	*D. concinna*	[72]
**178**	β-Sitosterol 3-*O*-β-D-glucopyranoside (daucosterol)	*D. draco*	[29]
**179**	β-Sitosterol 3-*O*-(6-*O*-palmitoyl)–β-D-glucopyranoside (sitoindoside I)	*D. draco*	[55]
**180**	Stigmasterol 3-*O*-β-D-glucopyranoside	*D. viridiflora*	[50]
**181**	1β-Hydroxy-kryptogenin 1-*O*-α-L-rhamnopyranosyl-(1→2)-α-L-arabinopyranoside	*S. cylindrica*	[60]

**Table 10:** Saponin patterns of *Dracaena* and *Sansevieria* species.

Species	Plant Parts Extracted	Saponins
*Dracaena afromontana* Mildbr.	methanolic extract of the twigs	**139**
*Dracaena angustifolia* Medik, (Roxb.)	methanolic extract of fresh stems; roots and rhizomes	**1,5,11,12,13,16,17,(23),28,29.35,37,39,50,51,53,72,(75),77,83,111,112,** **113,127,140,143,147a,168,175**
*Dracaena arborea* (Willd.) Link	methanolic extract of bark	**7,46,97,99,102**
*Dracaena cambodiana* Pierre ex Gagnep	fresh stems; dragon’s blood	**2,11,12,24,50,51,52,54,58,65,76,79,87,97,103,104,106,107,119,127,133, 164,165,168,173,176**
*Dracaena cochinchinensis* (Lour.) S.C. Chen	fresh stems (dragon’s blood)	**43,44,56,86,89,92,93,94,95,96,125,131,132,141,153,154,156,159,166,167,172,174**
*Dracaena concinna* Kunth	fresh stems	**28,36,82,85,88,117,136,137,138,144,160,163,177**
*Dracaena draco* L.	stem bark; aerial parts; leaves; roots	**7,11,14,15,17,18,19,20,21,25,26,45,47,49,53,60,85,88,101,124,133,155, 157,178,179**
*Dracaena fragrans* (L.) Ker Gawl. (syn. *D. deisteliana* Engl.)	methanolic extract of stems; bark, roots, leaves	**1,2,11,12,25,27,29,58,105**
*Dracaena mannii* Baker	fruit pulp; stem bark	**7,46,97**
*Dracaena marginata* Hort.	bark, roots	**6,28,30,32,40,41,77,81,90,128,129,150,158,162**
*Dracaena ombet* Heuglin ex Kotschy & Peyr.	leaves	**22,73,74**
*Dracaena surculosa* Lindl.	methanolic extract of whole plant	**8,9,10,31,45,48,55,101,120,121,122,123,135**
*Dracaena thalioides* Hort. Makoy ex E. Morren	leaves; fresh underground parts	**1,4,11,32,34,46,58,72,77,80,97,98,100,108,111,112,114,115,118,130,147,149**
*Dracaena viridiflora* Engl. & K. Krause	leaves	**6,25,26,85,88,161,180**
*Sansevieria cylindrica* Bojer ex Hook.	methanolic extract of unflowering aerial parts; leaves	**3,28,33,38,77,146,152,181**
*Sansevieria ehrenbergii* Schweinf. ex Baker	MeOH-CH_2_Cl_2_ extract of chipped plant	**25,42,57,84,88,91**
*Sansevieria trifasciata* Prain (syn. *D. trifasciata* (Prain) Mabb)	aerial parts; methanol extract of the whole plant	**11,58,59,60,61,62,63,64,65,66,67,68,69,70,71,72,77,78,79,107,108,109,** **110,111,114,116,126,134,142,145,148,151,168,169,170,171**

## Data Availability

Not applicable.

## Sample Availability

Samples of the compounds are not available from the authors.
